# Heavy Metal Content in Tattoo and Permanent Makeup Inks and European Standards—Is There Still a Health Risk?

**DOI:** 10.3390/toxics13110934

**Published:** 2025-10-30

**Authors:** Małgorzata Ćwieląg-Drabek, Joanna Furman, Klaudia Gut-Pietrasz

**Affiliations:** 1 Department of Environmental Health Risk Factors, Faculty of Public Health in Bytom, Medical University of Silesia in Katowice, 41-902 Bytom, Poland; jfurman@sum.edu.pl; 2 Department of Environmental Health, Faculty of Public Health in Bytom, Medical University of Silesia in Katowice, 41-902 Bytom, Poland; kgut@sum.edu.pl

**Keywords:** tattoo, permanent makeup, dermal exposure, risk assessment, heavy metals

## Abstract

Tattoos and permanent makeup involve intradermal pigment deposition and may introduce toxic trace elements into the body. Despite increasing popularity, harmonized EU regulations on tattoo ink composition only came into force in 2022 under REACH. This study evaluated the chemical safety of 41 commercially available inks in the EU following the implementation of these restrictions. Twelve heavy metals were analyzed (Cd, Pb, As, Hg, Zn, Cu, Ni, Cr, Co, Sb, Se, Mn). Copper showed the highest concentrations (mean 1751 mg/kg; max 25,701 mg/kg), while cadmium was lowest (mean 0.13 mg/kg). Exceedances of EU limits were recorded for Ni (24 samples), As (20), Cr(VI) (16), Cu (10), Sb (8), Co (6), and Pb (5); mercury was not detected in any ink. Dermal exposure was modeled across three tattooing scenarios using SED, MoS, HQ, and LCR indicators. Unacceptable non-cancer risk (MoS < 100) was mainly associated with copper (up to 85.4% of products), with additional concerns for zinc and arsenic (~50% of samples in higher-use scenarios). HQ values > 1 were most frequent for Ni, Cr(VI), and Cu, affecting up to 68.3%, 43.9%, and 58.5% of inks, respectively. Lifetime cancer risk above 1 × 10^−4^ was observed for nickel in several products. Despite recently tightened European regulations, a substantial share of inks remains non-compliant and may pose carcinogenic and non-carcinogenic health risks, underscoring the need for continued market surveillance and enforcement.

## 1. Introduction

Tattooing is a form of body art that involves inserting pigment into the skin using a needle or using other techniques, such as microblading, which inevitably cause damage to the skin barrier. The practice dates back thousands of years and has been used by various cultures around the world for ritual, spiritual, aesthetic, and social purposes. Today, tattoos are popular both as a form of artistic expression and as a way of expressing personal identity. Permanent makeup, also known as micropigmentation, is a technique used to insert pigments into the skin to create a long-lasting makeup effect. Although in its current form it is a relatively modern procedure, its roots date back to ancient times. With the growing popularity of tattoos and permanent makeup, there is also increasing interest in the chemical composition of tattoo inks and their impact on health. One of the important health issues is the presence of heavy metals in these products.

Although tattooing is increasingly widespread, scientific evidence regarding long-term toxicological risks remains limited. Most available data concern inks tested before the 2022 REACH restrictions were implemented, so it remains unclear whether current products comply with the updated EU safety requirements. Existing studies have largely focused on the presence of metals in inks rather than on internal exposure or potential health effects resulting from intradermal pigment deposition. Therefore, this study focuses specifically on regulated heavy metals in tattoo and permanent makeup (PMU) inks currently available on the European market and evaluates whether these products meet the newly implemented REACH standards. To our knowledge, this is the first investigation conducted after the enforcement of the 2022 restriction that not only quantifies metal contamination but also assesses dermal exposure and both carcinogenic and non-carcinogenic health risks using SED, MoS, HQ, and LCR indices. This approach enables a current, evidence-based evaluation of whether existing regulatory measures sufficiently protect consumer health.

### 1.1. The History of Body Tattooing

The art of tattooing has a rich and diverse history. From ancient cultures to modern societies, tattoos have been used for a variety of purposes, from ritualistic to aesthetic. As societies evolve, so does the practice of tattooing, adapting to changing values and trends. Tattoos were known in ancient Egypt, where they were used for religious and protective purposes. Archaeological finds, such as mummies from the Middle Kingdom period (c. 2040–1782 BC), show that tattoos were used by women, presumably as protection during pregnancy and childbirth. The designs included dots and lines arranged in geometric shapes. In Nubia, a region south of Egypt, tattoos had similar religious and ritual uses. The designs were often more complex and included symbols of deities and animals. In ancient China and Japan, tattoos had various meanings. In China, where they were known as ciqing, they were used as a sign of belonging to criminal groups or as punishment for criminals. In Japan, on the other hand, tattoos had ritual and aesthetic uses, especially during the Edo period (1603–1868), when a rich tradition of irezumi developed. Tattoos in Oceania have deep social and spiritual significance. In Polynesia, including Samoa and Hawaii, tattoos symbolized social status, courage, and tribal identity. This tradition continued for hundreds of years, and the tattooing process was a ritual marked by ceremonies. Among Native American tribes, such as the Inuit and tribes in the Great Plains, tattoos were used for protection, healing, and as symbols of personal achievement and social status. Designs included simple lines and intricate representations of animals and spirits. In medieval Europe, tattoos were relatively rare and were often associated with pagan or heretical practices. As Christianity spread, tattoos were often seen as incompatible with the Christian faith and were stigmatized. Interest in tattoos grew in Europe during the Age of Discovery, when European sailors such as Captain James Cook returned from Polynesia with tattoos. Tattoos began to be seen as an exotic art form, and in the 19th century, they became popular among sailors and the aristocracy, including European monarchs. In the 20th century, tattoos gained popularity among various subcultures, such as rock and roll, punk, and hip-hop. The 1970s and 1980s saw a resurgence of interest in tattooing as a form of contemporary art, which contributed to the development of new styles and techniques. Today, tattoos are widely accepted and practiced around the world, both as a form of art and personal expression. There are many contemporary tattoo styles, such as Realism, realistic tattoos that mimic photographs; Traditional American, a style characterized by thick outlines and a limited color palette; and Modern Geometry, tattoos consisting of symmetrical patterns and geometric shapes [1,2,3,4].

In ancient Egypt, both women and men used various forms of permanent makeup, using pigments to accentuate their eyes and lips. The use of charcoal and other natural dyes for permanent facial beautification demonstrates advanced knowledge of skin pigmentation. In India and the Middle East, henna was used to create permanent designs on the skin, which can be considered a form of permanent makeup, albeit with limited durability compared to today’s standards. Permanent makeup, as we know it today, began to develop in the 20th century. One of the pioneers of this technique was Georges Burchett, a British tattoo artist who, in the 1930s, introduced pigments into the skin to create the effect of permanent makeup. In the mid-20th century, permanent makeup gained popularity in the United States and Europe as a method of cosmetic enhancement. In the 1980s, micropigmentation techniques became more precise and safer thanks to advances in medical technology and a better understanding of the biological processes occurring in the skin [3,5].

### 1.2. The Use of Tattoos

Tattoos not only serve an aesthetic purpose but also have applications in medicine and scientific research. Tattoos are used for cosmetic reconstruction, for example, after mastectomy, where pigments are used to reconstruct the areola. Medical tattoos can also serve as markers in radiation therapy. Tattoo inks are also being studied for their potential applications in delivering drugs through the skin and as a tool for monitoring health (for example, through tattoos that change color in response to blood sugar levels) [6,7].

Contemporary permanent makeup techniques include a variety of procedures, such as microblading—a technique used to reconstruct eyebrows by introducing pigment under the skin using thin needles, which allows for a natural look of the hairs; permanent eyeliner—a procedure that allows for permanent contouring of the eyelids; and lip contouring—the use of pigments to emphasize the contour and fill the lips, resulting in fuller and more expressive lips. These techniques use advanced devices that allow for the precise introduction of pigments to a specific depth in the skin, minimizing the risk of infection and other complications. Permanent makeup is used not only for aesthetic purposes, but also for reconstructive purposes. It helps people who have lost their eyebrows or eyelashes due to diseases such as alopecia or cancer regain their natural appearance. These procedures are also used in the reconstruction of nipples after mastectomy [7,8].

### 1.3. The Popularity of Tattoos—Statistics

Body tattooing and permanent makeup have gained popularity over the past few decades. Statistics on body tattooing vary by region. Approximately 38% of people worldwide have at least one tattoo. In the United States, the percentage is high, at around 32%, with 22% of people having more than one tattoo [9]. Tattoos are also very popular in Europe, with the percentage of people with tattoos ranging from 12% to 24%, depending on the country. In some countries, such as Italy, as many as 48% of adults have a tattoo. Other countries, such as Sweden and Spain, also have high percentages (around 40%) [10]. The average age of people with tattoos in Europe is around 25–35, although tattoos are popular among various age groups. In recent years, there has been a noticeable increase in interest in tattoos, especially among younger generations. People with tattoos are most often between 20 and 40 years old, but tattoos are also popular among teenagers and older people. Tattoos are popular among both men and women. More and more women are deciding to get tattoos, and in some age groups (e.g., 20–30 years old), the percentage of women with tattoos is higher than that of men. The most common places for tattoos are the forearms, shoulders, back, and legs. Many people also choose to get tattoos on their chest, wrists, or ankles. In recent years, tattoos on the neck and hands have also grown in popularity. Tattoos are becoming more and more accepted in various social and professional groups, including corporations. Most people who decide to get a tattoo do so for personal, aesthetic, or symbolic reasons. Tattoos have become part of mass culture and are now considered a form of artistic and personal expression. This trend is likely to continue, and the number of people with tattoos will grow both in Europe and worldwide [11,12]. Permanent makeup, a form of cosmetic tattooing, is also growing in popularity, especially among women. More and more women, but also men, worldwide are considering or have already undergone permanent makeup treatment, reflecting the growing acceptance and demand for long-lasting aesthetic solutions [13,14].

### 1.4. Composition of Tattoo Inks

Tattoo inks consist of pigments, which provide color, and carriers, which enable their application. These ingredients should be safe for health. Pigments used in tattoo inks can be organic or inorganic. Examples of organic pigments include carbon compounds such as azo pigments, while inorganic pigments include metal oxides such as iron oxide (Fe_2_O_3_) used to produce red and brown shades, and titanium dioxide (TiO_2_) for white. Carriers are substances that help distribute pigments evenly and allow them to penetrate the skin. The most commonly used carriers are distilled water, glycerin, ethanol, and propanol. These carriers are intended to stabilize the pigment and prevent infection. Tattoo inks may also contain additives such as stabilizers, preservatives, or pH regulators to ensure product durability and safety. In the past, pigments sometimes contained heavy metals due to their intense coloring properties. Lead (Pb) was used in some pigments, especially red and yellow colors; cadmium (Cd) in yellow pigments; chromium (Cr) in green and black inks; mercury (Hg) in red pigments; and nickel (Ni) often appeared in green and black pigments; and cobalt was used in blue pigments [15,16].

### 1.5. Safety of Tattoo Ink Use—Potential Health Risks

Tattoos have become an integral part of contemporary culture, serving as a form of artistic expression and personal statement. However, the tattooing procedure is associated with various side effects and potential health risks. Tattooing involves inserting ink under the skin using a needle that pierces the epidermis and introduces pigment into the dermis. Although generally considered safe, this process carries the risk of various side effects. The safety of tattoo ink is the subject of research and regulation around the world. While some ingredients are considered safe, others may cause allergic reactions, irritation, or other adverse health effects. The presence of heavy metals in tattoo ink poses a variety of health risks, both short- and long-term. Some individuals may experience allergic reactions to certain pigments, especially those containing nickel, cobalt, or chromium. Symptoms may include itching, redness, and swelling. Long-term exposure to heavy metals can lead to the accumulation of these substances in the body, which can result in serious health problems, including damage to internal organs, neurotoxicity, and an increased risk of cancer. Some heavy metals, such as chromium and cadmium, are considered carcinogenic. Their presence in tattoo inks raises concerns about a potential increase in the risk of developing skin cancer and other types of cancer. It is worth noting that improper sterilization of equipment or the use of ink containing microbial contaminants can lead to skin infections [17,18].

### 1.6. Legal Regulations

Due to the potential health risks associated with heavy metals in tattoo inks, many countries are implementing regulations to limit their content and ensure user safety. In the European Union, tattoo inks are regulated under the REACH (Registration, Evaluation, Authorization, and Restriction of Chemicals) Regulation, which restricts the use of certain chemicals in cosmetics. REACH requires the registration and evaluation of chemicals used in inks. New regulations that came into force in January 2022 set limits on the content of certain heavy metals and other chemicals in tattoo inks [19,20] (Table 1).

In the United States, the FDA (Food and Drug Administration) monitors the safety of tattoo inks, although they are not approved in the same way as other cosmetic products [21]. In the US, there is a need for more restrictive regulations on the composition of tattoo inks to ensure their safety. In many other countries, regulations on tattoo inks are less restrictive or non-existent. As awareness of the risks associated with heavy metals grows, many countries are moving to develop and implement appropriate regulations.

The introduction of certain amounts of chemical compounds, including toxic heavy metals, which are found in pigments used in tattoo ink and permanent makeup, directly under the skin, may pose a significant health risk, especially since it mainly affects young people. Because pigments are permanently deposited into the dermis, exposure to contaminants may be continuous and systemic, unlike other consumer chemical exposures, where contact is temporary. Young adults form the highest share of tattoo users, making long-term toxicological evaluation essential.

The study aimed to analyze the content of selected heavy metals (Cd, Pb, As, Hg, Cr, Cu, Co, Ni, Mn, Sb, Se, Zn) in tattoo inks available on the European market after REACH 2022 implementation in terms of their harmful effects on the human body. Chemical analysis of tattoo inks available on the European market has enabled the assessment of health risks associated with the introduction of toxic heavy metals into the body and the identification of products with the highest concentrations of the analyzed metals. The study aims to assess whether the tattoo and permanent makeup inks supplied by identified, easily accessible, common, or commercially established brands of tattoo ink suppliers (listed in Table S1—Supplementary Materials), which must comply with European standards, still pose a real threat to human health.

## 2. Materials and Methods

### 2.1. Collection of Research Material

The research material included in the study covered samples of tattoo inks and permanent makeup available on the European market. A total of 41 samples were analyzed for heavy metal content, of which 27 were tattoo ink samples, 9 were ink samples dedicated to tattooing and permanent makeup, and 5 were permanent makeup samples. Although this number provides a broad overview of the products available on the European market, it also represents a limitation and does not allow for complete statistical generalization. The samples were selected using a convenience-based strategy, prioritizing commercial availability and market popularity. The inks were obtained from seven manufacturers, representing the most frequently distributed brands in professional tattoo studios and on major European online platforms. Of the analyzed inks, 88% were manufactured in the United States, while the remainder were of Chinese or European origin. However, all products were purchased within the EU (mainly via EU-based distributors and online stores) and were therefore considered representative of items available to European consumers. Purchases were made between 2022 and 2023, shortly after the entry into force of the REACH restrictions concerning tattoo and permanent makeup inks. Most products were labeled in English and included batch numbers, ingredient lists, and manufacturer details. For some products, information confirming compliance with EU/REACH requirements was found, although this information was not provided on the packaging, but was only found on the manufacturer’s website. Given the diversity of manufacturers, colors, and intended uses, the selected sample set provides an informative cross-section of inks available and used on the European market, while also acknowledging that not all brands or formulations could be included. The products were purchased in their original, factory-sealed packaging. The samples were stored in original packaging and kept at room temperature (23 °C) until they were analyzed. The study covered both black and colored inks (red, orange, yellow, white, blue, green, brown, pink, purple).

### 2.2. Laboratory Analyses

After shaking each package, approximately 0.1 g of ink (±0.005) was measured directly into a Teflon vessel. The collected test material was then subjected to microwave mineralization using a Magnum II mineralizer (ERTEC, Wrocław, Poland) and perchloric acid (HClO_4_—3 mL), hydrochloric acid (HCl—1 mL), and hydrofluoric acid (HF—1 mL)—purchased from Merck Company (Darmstadt, Germany). Deionized water (with analytical grade) was obtained using a Polwater DL2-100 deionizer from Labopol-Polwater Company (Kraków, Poland). In mineralized samples, zinc, chromium, nickel, copper, arsenic (inorganic), cobalt, antimony, selenium, and manganese concentrations were determined using inductively coupled plasma optical emission spectrometry (ICP-OES; Ultima Expert LT, Horiba Scientific, Palaiseau, France). Cadmium and lead concentrations were determined using the electrothermal atomic absorption spectrometry (ET-AAS; Savanta Sigma, GBC, Melbourne, Australia) method, while mercury was determined using a mercury analyzer (Millenium Merlin, PS Analytical, Orpington, Kent, UK), utilizing the cold vapor generation technique in combination with atomic fluorescence spectroscopy (AFS). Laboratory analyses were performed in the Analytical Laboratory of the Department of Environmental Health, Faculty of Public Health in Bytom, of the Medical University of Silesia in Katowice, accredited by the Polish Center for Accreditation.

To ensure measurement consistency, certified reference solutions with a concentration of 1000 mg/l in HNO_3_ solution were used to determine the calibration curves for individual elements and to verify the correctness of the measurements: Zn–Accu Standard (220065027), Cr–Accu Standard (220075097), Ni–Agilent Technologies (0104978089), Cu–Accu Standard (221075081), As–Agilent Technologies (0110371350), Co–SCP Science (S210514050), Sb–SCP Science (S210505011), Se–Agilent Technologies (0106315884), Mn–SCP Science (S210526015), Cd–Accu Standard (ICP-08N-1 220065069), Pb–SpexCerti Prep. (26-71 PBT), Hg–Accu Standard (ICP-34-N-1). The accuracy of the results obtained was assessed by analyzing samples with the addition of a standard of known concentration. The average recovery values are presented in Table 2. In order to determine the limits of detection (LOD) and quantification (LOQ) for individual elements, 20 mineralized blank samples were analyzed. For each series, analytical signal measurements were performed, the standard deviation of the obtained values was calculated, and then the following formulas were used:

(1) $$LOD=\frac{mean\;value\;of\;zero\;sample\;signals}{3\times standard\;deviation}$$

(2) $$LOQ=\frac{mean\;value\;of\;zero\;sample\;signals}{6\times standard\;deviation}$$

The obtained LOQ (Limit of Quantification) values were as follows: Pb—0.80 mg/kg, Cd—0.08 mg/kg, Zn—7.80 mg/kg, Cr—3.68 mg/kg, Ni—7.40 mg/kg, Cu—5.03 mg/kg, As—10.95 mg/kg, Co—3.30 mg/kg, Sb—11.58 mg/kg, Se—14.00 mg/kg, Mn—2.35 mg/kg, Hg—0.008 mg/kg. All data concerning the method (spectrometer measurement conditions and calibration parameters) are presented in Table 3.

### 2.3. Assessment of Dermal Health Risks from Heavy Metals Contained in Tattoo and Permanent Makeup Inks

Tattoo ink usage depends on many technical factors—there is no single fixed value in grams that would be necessary to cover, for example, 1 cm^2^ of skin. Factors affecting ink consumption:

- Tattoo artist technique—some artists use less pigment, others use more;
- Needle type—thin needles for contouring use minimal amounts, wide needles (e.g., for filling or shading) use much more;
- Type of work—lines vs. full color filling;
- Color—black pigments often cover better and require fewer layers than, for example, light colors;
- The skin of the person being tattooed—elasticity, moisture, location on the body.

In tattooing practice, it is generally assumed that a small 5 × 5 cm (25 cm^2^) tattoo usually requires about 0.5–1.5 mL of ink, or approx. 0.5–1.5 g (ink has a density close to that of water). This means that an average of 0.02–0.06 mL (≈0.02–0.06 g) of ink is needed per 1 cm^2^ of skin. Table 4 shows the estimated ink consumption (min/typical/max) calculated for areas of 5, 10, and 100 cm^2^ in three styles: contour, filling, and realism. A simple conversion of 1 g ≈ 1 mL was assumed. The estimates were derived from consultations with professional tattoo artists and cross-checked with manufacturer-reported pigment use per surface area from technical datasheets.

Further calculations took into account ink usage for a 5 cm^2^ tattoo, considering minimum, typical, and maximum ink consumption in three tattooing styles (contour, realism, and filling).

Because ICP-OES does not allow chromium speciation to be determined, all analytical measurements reflect total chromium (Cr). However, regulatory toxicity thresholds and reference values relevant to tattoo exposure (e.g., REACH limitations) apply exclusively to hexavalent chromium (Cr(VI)). To avoid overestimating health risks, a conservative assumption was applied in all health risk calculations: 1% of the quantified total chromium was considered to be present as Cr(VI). Therefore, while Table S2 presents the measured total chromium concentrations along with LOD/LOQ values (Table 2), the risk assessment metrics (MoS, HQ, and LCR) were calculated using only 1% of total Cr, treated as Cr(VI).

The health risk to humans resulting from exposure to heavy metal contaminants present in tattoo inks and permanent makeup was calculated in relation to the margin of safety (MoS), which can be estimated by considering the ratio of the no observed adverse effect level (NOAEL) of the tested product to its systemic exposure dose (SED) [22], according to Equation (3):

(3) $$MoS=\frac{NOAEL}{SED}$$

where MoS—margin of safety [unitless value]; NOAEL—no observed adverse effect level [mg/kg body weight/day]; SED—systemic exposure dose [mg/kg body weight/day]. MoS refers to the systemic risk of toxic effects, i.e., risks of chronic exposure—long-term, low doses, e.g., in cosmetics or food; risks of side effects—e.g., teratogenic effects (on the fetus), organ toxicity; risks of consumer exposure—determines whether the use of a given product is safe for humans. Interpretation of MoS values [23]:

- MoS ≥ 100—acceptable safety level—it is assumed that there is a sufficient margin of protection, taking into account interspecies variability (animals–humans) and intraspecies variability (differences between humans). The higher the MoS value, the greater the margin between the exposure level and the level that may cause harm. A safety margin of at least 100 is generally considered sufficient to protect humans from the toxic effects of a chemical if the NOEL value is based on toxicological data from animal studies.
- MoS < 100—may indicate potential health risks and the need for further analysis or exposure reduction.

SED estimates the amount of chemicals that enter the human body through various routes of exposure. It is calculated based on the concentration of the element present in the tested product, the amount of product used per day, the frequency of use, the skin area to which the product is applied, and the average body weight [22]. The SED value was calculated using Equation (4):

(4) $$SED=\frac{C\times AA\times SSA\times RF\times DAF}{BW}\;\times 10^{-3}$$

where C—concentration of the element determined in the analyzed ink sample [mg/kg]; AA—amount applied [g/cm^2^] (adopted in accordance with Table 2); SSA—skin surface area [cm^2^] (adopted in accordance with Table 2); RF—retention factor [unitless value] (1 adopted); DAF—dermal absorption factor [%] (the following values were set: for Cd, Cr, Cu & Sb—0.001%, for Se & Mn—0.01%, for Pb—0.003%, Zn—0.02%, Ni—0.04%, As—0.03%, Co—0.8%); BW—body weight [kg] (60 kg assumed); 10^−3^—unit conversion factor (mg/kg). The dermal absorption factors (DAFs) used in this study were adopted from previously published data [24,25,26] and are consistent with values commonly applied in human health risk assessment frameworks (e.g., U.S. EPA, ATSDR). The variation among elements (0.001–0.8%) reflects differences in their physicochemical properties, solubility, and affinity for skin penetration. For instance, very low values were applied for Cd, Cr, Cu, and Sb (0.001%) due to their low dermal permeability, while higher values were assigned to more mobile or bioavailable elements such as Co (0.8%) and Ni (0.04%) based on experimental and modeled data. It should be noted that DAF values reported in the literature may vary by several orders of magnitude depending on experimental conditions (e.g., exposure medium, pH, or species of the element). Therefore, the uncertainty associated with these assumptions may influence the absolute values of calculated dermal exposure, although the overall contribution of dermal contact to total exposure remains typically low compared to ingestion and inhalation pathways.

The exposure level at which no adverse health effects are observed, referred to as NOAEL, is calculated based on reference doses (RfD) determined for the dermal route [22] using the following Equation (5):

(5) $$NOAEL={RfD}_{dermal}\times UF\times MF$$

where RfD_dermal_—reference dose [mg/kg] (calculated based on the reference dose set for the ingestion route and dermal absorption factor (DAF) in accordance with Equation (4); UF—uncertainty factor reflecting overall confidence in various data sets [unitless value] (the following values were set: 1 for Mn, 3 for Zn, As & Se, 10 for Cd & Cu, 100 for Pb, Cr & Co, 300 for Ni, 1000 for Sb [27]; MF—modifying factor (the value 1 was adopted).

Calculation of reference doses (RfD) for the dermal route [28]:

(6) $${RfD}_{dermal}={RfD}_{ingestion}\times DAF$$

where RfD_ingestion_—reference dose [mg/kg/day] for oral exposure (adopted values: 0.001 for Cd, 0.004 for Pb, 0.3 for Zn, 0.0009 for Cr(VI), 0.02 for Ni, 0.04 for Cu, 0.06 for As, 0.03 for Co, 0.0004 for Sb, 0.005 for Se, and 0.14 for Mn) [27]. Final RfD values adopted for the dermal route: 1.0 × 10^−6^ for Cd, 1.2 × 10^−5^ for Pb, 6.0 × 10^−5^ for Zn, 9.0 × 10^−7^ for Cr(VI), 8.0 × 10^−4^ for Ni, 4.0 × 10^−5^ for Cu, 1.8 × 10^−3^ for As, 2.4 × 10^−2^ for Co, 4.0 × 10^−7^ for Sb, 5.0 × 10^−5^ for Se, and 1.4 × 10^−3^ for Mn.

The hazard quotient (HQ; unitless value), calculated to estimate non-carcinogenic health risk, is the ratio of the systemic exposure dose (SED) to the dermal reference dose (RfD) set for the element. An HQ value less than or equal to 1 is considered safe, while a value greater than 1 carries a risk of non-cancerous health effects. The HQ level was calculated using the following Equation (7) [22]:

(7) $$HQ=\frac{SED}{{RfD}_{dermal}}$$

In the study, the lifetime cancer risk (LCR; unitless value) was also estimated for carcinogenic elements using Equation (8) [22]:

(8) $$LCR=\frac{SED}{{CSF}_{dermal}}$$

where CSF_dermal_ is a cancer slope factor [mg/kg] assigned to a given element via dermal exposure. CSF approximates the risk of developing cancer per unit dose of a substance considered carcinogenic over a specified lifetime. It determines the slope of the curve between dose and cancer risk. CSF was calculated using Equation (9) [29]:

(9) $${CSF}_{dermal}=\frac{{CSF}_{ingestion}}{DAF}$$

where CSF_ingestion—_cancer slope factor designated for a given element for oral exposure [mg/kg]. The study adopted the following CSF_ingestion_ values for carcinogenic elements: 0.0085 for Pb, 0.38 for Cd, 0.27 for Cr(VI), 1.7 for Ni, and 32 for As. The final CSF values adopted for the dermal route: 3 for Pb, 380 for Cd, 270 for Cr(VI), 43 for Ni, and 1067 for As.

LCR is a key indicator used to estimate the cancer risk associated with exposure to a given chemical substance throughout a person’s lifetime. LCR is the probability that a person will develop cancer as a result of long-term (chronic) exposure to a given substance. It is based on the estimated exposure (SED) and the carcinogenic potential of a given substance (CSF). Acceptable cancer risk levels vary depending on the country and institution. However, the following thresholds are most commonly used [29]: <1 × 10^−6^—no significant risk (acceptable), 1 × 10^−6^ to 1 × 10^−4^—potentially acceptable depending on the context (e.g., low exposure, lack of alternatives), >1 × 10^−4^—unacceptable risk; may require exposure reduction measures. A level > 1 × 10^−4^ is interpreted as meaning that more than one person in 10,000 with the same exposure will develop cancer during their lifetime.

Values in the tables in the Results section that did not fall within the acceptable range set for MoS (<100), HQ (>1), and LCR (>1 × 10^−4^) are marked in bold; in the case of Supplementary Materials, values exceeding the norm are marked in red.

### 2.4. Statistical Analyses

Data processing and statistical analysis were completed using Excel 2024 and Statistica 13 software (StatSoft, Kraków, Poland). The distributional properties of the analyzed variables were first assessed to verify the assumption of normality. The results indicated that the data did not follow a normal distribution. Consequently, non-parametric statistical methods were applied. To compare differences between groups, the Kruskal–Wallis analysis of variance (ANOVA) test was employed, as it does not require the assumption of normally distributed data.

## 3. Results

### 3.1. Heavy Metal Concentrations in Analyzed Samples of Tattoo and Permanent Makeup Inks

Of the 12 elements identified in the tested samples of tattoo and permanent makeup inks, none of the 41 samples exceeded the LOQ (0.008 mg/kg) for mercury; more precisely, no Hg was detected in any of the samples analyzed. Table S2 shows only concentrations exceeding the LOQ set for individual elements. When analyzing these results, the highest average concentration of an element was obtained for copper (1751.01 mg/kg), while the lowest was for cadmium (0.13 mg/kg). Three samples had selenium concentrations (average 18.49 mg/kg) exceeding the LOQ, five samples had lead concentrations (average 2.81 mg/kg) > LOQ, six samples had cobalt concentrations (average 44.14 mg/kg) > LOQ, antimony in eight samples (average 16.03 mg/kg), manganese in nine samples (average 169.13 mg/kg), cadmium in ten samples, zinc in twenty samples (average 38.81 mg/kg) and arsenic in twenty samples (average 20.06 mg/kg), twenty-four samples for nickel (average 62.98 mg/kg), thirty-five for copper, and forty-one for chromium (average 58.97 mg/kg) (Table S2 and Table 5). Of the 41 samples analyzed, the highest number of elements exceeding the LOQ was found in tattoo ink sample No. 11, with 10 elements; 9 elements were found in samples No. 2 and 27 (both tattoo ink samples); each sample came from a different manufacturer. Only one element (chromium), whose concentration exceeded the LOQ, was found in samples No. 4 and 10 (both samples were tattoo inks from the same manufacturer). Table 5 presents descriptive statistics of the distribution of concentrations of the analyzed elements in all samples of inks included in the study. Considering the intended use of the inks (tattoo, permanent makeup, tattoo & permanent makeup), no Pb concentrations exceeding the LOQ for this element were found in inks intended for both tattooing and permanent makeup. No concentrations of Co, Sb, Se, and Mn exceeding the LOQ were also found in this product group. The highest average concentration recorded in this product group was for copper (1967.05 mg/kg), and the lowest was for cadmium (0.10 mg/kg). In the group of permanent makeup products, no concentrations exceeding the limit of quantification (LOQ) were recorded for As, Sb, and Se. The highest average concentration of an element in this product group was recorded for manganese (average 441.78 mg/kg), which was influenced by the Mn concentration recorded in sample No. 28 (1089.81 mg/kg), while the lowest was for cadmium (0.18 mg/kg). In the group of tattoo inks, the highest average concentration was recorded for copper (2058.45 mg/kg), while the lowest was for cadmium (0.09 mg/kg). When analyzing the results in terms of ink color (excluding inks dedicated exclusively to permanent makeup), it should be noted that the color with the highest number of elements whose concentrations exceeded the LOQ was brown. This was followed by yellow, pink, white, blue, orange, white, purple, black, and green. The lowest number of elements whose concentration exceeded the LOQ was recorded in red ink samples. Four tattoo ink packages from one manufacturer bore the statement “May contain trace amounts of Nickel.” Of these four samples, Ni concentrations above the LOQ were recorded in three.

Concerning the normative values applicable in the European Union [20], listed in Table 1, concentrations exceeding the limits were recorded for nickel in 24 samples, arsenic in 20 samples, chromium (VI) in 16 samples (assuming the accepted estimates for Cr(VI)), copper in 10 samples, antimony in 8 samples, cobalt in 6 samples, lead in 5 samples, and selenium in 3 samples. The standards set for mercury, cadmium, and zinc were not exceeded in any of the samples. No such standards have been set for manganese. In the case of ink samples intended for permanent makeup, two (from the same manufacturer) of the five analyzed in the study exceeded the values set for Pb. In all (*n* = 5) samples, the Ni concentration exceeded the permissible limit. In two samples, the limit set for Co was exceeded, and in two others, the limit set for Cr (VI). When analyzing a group of tattoo inks in three samples, the limit set for Pb (each of a different color) and Se (two inks were brown) was exceeded; in the case of Cr (VI), the limit was exceeded in 12 samples (mainly orange and pink inks), 8 samples when analyzing the concentrations of Cu (mainly green and blue inks) and Sb (mainly pink and brown inks), 4 samples when considering the limit for Co (two inks were brown), and all samples when analyzing the permissible limit set for nickel and arsenic. In all ink samples (those for which the concentration of the element was >LOQ) intended for both tattooing and permanent makeup, concentrations of nickel and arsenic exceeding the permissible limit were recorded; in two samples, exceedances were recorded for Cr (VI) concentrations, and in another two for Cu.

### 3.2. Statistical Analysis Results

**Figure 1:**
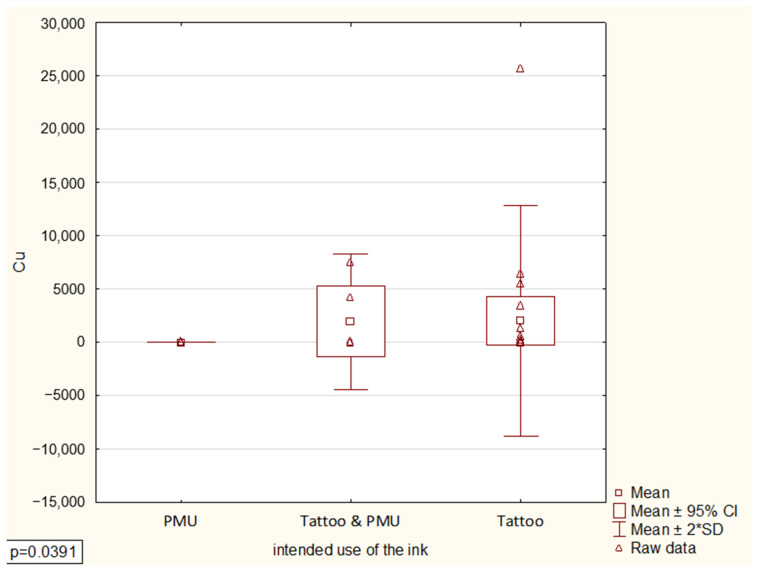
Box-and-whisker plot of copper concentration by intended use of ink (Kruskal–Wallis *p* = 0.0391) (The asterisk represents the multiplication sign).

The statistical analysis assessed the relationships between the concentration of the tested element and the intended use of the ink, its color, and the manufacturer. The only statistically significant relationship was found in the Kruskal–Wallis ANOVA test for multiple independent samples, specifically the relationship between copper concentration and the intended use of the ink (*p* = 0.0391) (Figure 1). Further multiple comparisons of mean ranks showed that tattoo inks had a statistically significantly higher mean rank of copper concentration compared to permanent makeup inks. Copper pigments are more prevalent in tattoo inks (particularly blue, green, and brown colors) than in PMU inks, which mainly use organic pigments. This probably explains the significant variation observed. However, it is essential to note that due to the limited number of samples in this product group (permanent makeup inks), the results should be interpreted with caution. Other metals showed high within-group variability and small subgroup sizes, which may have masked additional statistical relationships.

### 3.3. Non-Cancer Health Risk

The study assumed the estimation of non-cancer health risks based on SED, MoS, and HQ, focusing on three main exposure scenarios. Exposure was calculated for a 5-cm tattoo made using the contouring method (scenario 1), the realistic method (scenario 2), and the filling method (scenario 3). Contouring, also called outlining, is the first stage of most tattoos. The artist uses a tattoo machine with a fine grouping of needles to draw clean, precise lines that define the design. The realistic method focuses on creating depth and lifelike effects through shading. Instead of solid lines, the artist builds up tones gradually, using smooth gradients from dark to light. Filling is the technique of saturating large areas with solid ink. The artist works in circular or back-and-forth motions to evenly distribute pigment, ensuring that no gaps or light spots remain. For each method (according to the data in Table 4), three further sub-scenarios were calculated, taking into account ink usage: minimum, typical, and maximum.

Data on systemic exposure dose (SED) are provided in Tables S3–S5 (Supplementary Materials). Based on SED, the margin of safety (MoS) was calculated—individual results for each of the analyzed inks are included in Tables S6–S8 (Supplementary Materials). Table 6 summarizes the results presented in Tables S6–S8, indicating for how many samples the MoS value was below 100, taking into account the tattooing method and ink usage. When analyzing all the elements tested, a MoS value below 100 was mainly observed for copper, affecting 75.6% of samples in the scenario assuming minimum ink consumption during tattooing/permanent makeup using the contouring method, as well as 85.4% of samples in the other scenarios. MoS values < 100 at a level of approximately 50% of the samples tested were also recorded for the scenario assuming typical and maximum ink usage in the realistic and filling method—this applied to elements such as zinc and arsenic (Table 6). When analyzing the colors of the inks tested in terms of the number of elements for which the MoS value was below 100, brown and black performed the worst, but yellow and pink also performed poorly (Tables S6–S8). When comparing inks in terms of their intended use, taking into account the average MoS values calculated for individual elements in all analyzed scenarios, excessive values (MoS < 100) in the group of tattoo inks were recorded for Cd, Zn, Cu, As, Sb, Se, Mn, in the group of inks dedicated to permanent makeup, these were Cd, Zn, Cr, Ni, Cu, Co, Mn, and for the last group—tattoo and permanent makeup inks—Cd, Zn, Ni, Cu, As (Table 7).

Tables S9–S11 (Supplementary Materials) contain data presenting the estimated HQ values for individual ink samples, analogous to those for MoS (taking into account the tattooing method and ink consumption). HQ values exceeding 1 were recorded mainly for chromium (VI), nickel, and copper (Table 6). In the scenario involving tattooing/permanent makeup using the filling method, assuming the highest ink usage, the permissible value was exceeded in 43.9, 58.5, and 68.3 percent (%) of samples, respectively. When analyzing the average HQ values, estimated taking into account the division by ink purpose, tattoo ink samples recorded HQ values exceeding 1 for elements such as Cr (VI), Ni, Cu, Co, Sb, Se, Mn, in the case of permanent makeup inks, these were Pb, Cr, Ni, Cu, Co, Mn, and in those dedicated to tattooing and permanent makeup—Cr, Ni, and Cu (Table 8). Considering the colors of the inks tested, the largest number of elements for which the estimated HQ exceeded 1 concerned brown inks, but also black and yellow inks (Tables S9–S11).

Overall, copper (Cu), nickel (Ni), chromium (Cr), and arsenic (As) consistently represent the most significant toxicological concern across all exposure scenarios, for all the analyzed product types, as demonstrated by low MoS and high HQ values. Interpretation of the non-carcinogenic risk for each scenario indicates that:

- Scenario 1 (contouring): The highest non-cancer risk is associated with copper, affecting most samples even at minimum ink usage. Zinc and arsenic also pose a moderate risk in typical and maximum consumption sub-scenarios.
- Scenario 2 (realistic): Risk increases for zinc, arsenic, and copper, particularly in typical and maximum ink usage, reflecting the cumulative effect of shading techniques.
- Scenario 3 (filling): Saturation of large areas amplifies systemic exposure to nickel, chromium, and copper, leading to HQ values exceeding 1 in a substantial proportion of samples.

In summary, while all methods show potential non-carcinogenic risks, the filling method generally produces the highest exposure, highlighting the need to control the use of Cu, Ni, Cr, and As in tattoo and permanent makeup inks. These elements should be prioritized in regulatory and risk mitigation strategies.

### 3.4. Cancer Health Risk

Tables S12–S14 (Supplementary Materials) present LCR values calculated for lead, cadmium, chromium (VI), nickel, and arsenic. These tables list values ranging from 1 × 10^−6^ to 1 × 10^−4^ (i.e., potentially acceptable) and >1 × 10^−4^ (unacceptable risk). LCR results calculated for nickel were recorded within the potentially acceptable range—in each scenario analyzed, this concerned samples for which the concentration of this element exceeded the LOQ, with LCR values > 1 × 10^−4^ in this group. In the first scenario, which took into account the use of the contouring method, the acceptable LCR level was exceeded in 2 samples, taking into account typical ink consumption, and in 8 samples at maximum consumption. In the second scenario (realistic method), there were two samples with minimum ink usage, eight with typical usage, and eleven with maximum usage. In the third scenario, there were 8, 11, and 12 samples, respectively (Table 6). An LCR value of 1 × 10^−6^ to 1 × 10^−4^ was also recorded for lead—in the scenario using the filling technique, assuming maximum ink usage, a total of 4 samples (2 tattoo ink samples and 2 permanent makeup ink samples) fell within this range (Table S14). When analyzing the average LCR values obtained for individual exposure scenarios, the values obtained for nickel were >1 × 10^−5^ in each scenario (Table 9). Values > 1 × 10^−4^ were recorded in scenarios 2 (realism) and 3 (filling) for products dedicated to tattooing and permanent makeup, which took into account maximum ink consumption in scenario 2 and typical and maximum consumption in scenario 3. In scenarios involving tattoo products and permanent makeup products, LCR values of 1 × 10^−6^ were also recorded (Table 9). The ink color for which an LCR value of >1 × 10^−4^ was recorded in the method taking into account contouring (with typical ink usage) was pink ink dedicated to tattooing (Table S12). The same result (>1 × 10^−4^) was obtained for purple, yellow, and orange ink, but in the method taking into account realism, while for pink ink, this level was recorded in this method already in the scenario taking into account minimum ink usage (Table S13). In this method, LCR > 1 × 10^−4^ was also recorded for two permanent makeup inks (from the same producer) (Table S13), where for one of them (brown-black color), this level was already recorded at minimum ink usage. In the scenario assuming the filling method (Table S14), the LCR value estimated for Ni at × 10^−4^ was already recorded at minimum ink usage for blue, purple, black, orange, and pink ink—all products were dedicated to tattooing, and for the two previously indicated permanent makeup inks. The interpretation of the carcinogenic risk (LCR) for individual scenarios can be summarized as follows:

- Scenario 1 (contouring): LCR values exceeding 1 × 10^−4^ were observed sporadically, mainly in pink tattoo inks at typical consumption, indicating a limited but notable carcinogenic risk.
- Scenario 2 (realistic): The carcinogenic risk increases with ink usage, particularly in purple, yellow, and orange tattoo inks, as well as select permanent makeup inks. This scenario demonstrates that shading techniques can lead to higher exposure to nickel and other carcinogenic elements.
- Scenario 3 (filling): The highest LCR values are observed across multiple ink colors, including blue, purple, black, orange, and pink, even at minimum ink usage, underscoring the elevated cancer risk associated with saturation techniques.

Overall, nickel consistently emerges as the primary carcinogenic concern across all scenarios, contributing variably depending on ink color, type, and application method. The identification of this priority contaminant underscores the urgent necessity for strict monitoring and compliance, as well as enhanced consumer education. These findings support targeted regulatory actions and consumer guidance to reduce potential carcinogenic exposure from tattooing and permanent makeup products.

**Table 8:** Average HQ (Hazard Quotient) values obtained for the analyzed samples of tattoo and permanent makeup inks by type of tattoo performed.

**Ink Type/Element**	**Contour**			**Realism**			**Filling**		
**Tattoo**	**MIN**	**TYPICALLY**	**MAX**	**MIN**	**TYPICALLY**	**MAX**	**MIN**	**TYPICALLY**	**MAX**
Pb	0.040	0.081	0.121	0.081	0.161	0.242	0.121	0.242	0.362
Cd	0.032	0.064	0.095	0.064	0.127	0.191	0.095	0.191	0.286
Zn	0.043	0.085	0.128	0.085	0.171	0.256	0.128	0.256	0.384
Cr	0.229	0.459	0.688	0.459	0.917	**1.376**	0.688	**1.376**	**2.063**
Ni	**1.218**	**2.437**	**3.655**	**2.437**	**4.873**	**7.310**	**3.655**	**7.310**	**10.965**
Cu	**18.379**	**36.758**	**55.137**	**36.758**	**73.516**	**110.274**	**55.137**	**110.274**	**165.411**
As	0.013	0.026	0.039	0.026	0.051	0.077	0.039	0.077	0.116
Co	0.574	**1.149**	**1.723**	**1.149**	**2.298**	**3.446**	**1.723**	**3.446**	**4.136**
Sb	**1430.843**	**2861.685**	**4292.528**	**2861.685**	**5723.371**	**8585.056**	**4292.528**	**8585.056**	**12,877.584**
Se	**66.046**	**132.092**	**198.138**	**132.092**	**264.184**	**396.275**	**198.138**	**396.275**	**594.413**
Mn	**4.184**	**8.368**	**12.552**	**8.368**	**16.736**	**25.103**	**12.552**	**25.103**	**37.655**
**Ink type/Element**	**Contour**			**Realism**			**Filling**		
**Makeup**	**MIN**	**TYPICALLY**	**MAX**	**MIN**	**TYPICALLY**	**MAX**	**MIN**	**TYPICALLY**	**MAX**
Pb	0.149	0.298	0.447	0.298	0.596	0.893	0.447	0.893	**1.340**
Cd	0.064	0.127	0.191	0.127	0.254	0.381	0.191	0.381	0.572
Zn	0.078	0.157	0.235	0.157	0.314	0.471	0.235	0.471	0.706
Cr	0.335	0.670	**1.006**	0.670	**1.341**	**2.011**	**1.006**	**2.011**	**3.017**
Ni	**1.312**	**2.624**	**3.935**	**2.624**	**5.247**	**7.871**	**3.935**	**7.871**	**11.806**
Cu	0.143	0.286	0.430	0.286	0.573	0.859	0.430	0.859	1.289
As	-	-	-	-	-	-	-	-	-
Co	0.427	0.855	**1.282**	0.855	**1.710**	**2.565**	**1.282**	**2.565**	**3.847**
Sb	-	-	-	-	-	-	-	-	-
Se	-	-	-	-	-	-	-	-	-
Mn	**56.349**	**112.698**	**169.048**	**112.698**	**225.397**	**338.095**	**169.048**	**338.095**	**507.143**
**Ink type/Element**	**Contour**			**Realism**			**Filling**		
**Tattoo & Makeup**	**MIN**	**TYPICALLY**	**MAX**	**MIN**	**TYPICALLY**	**MAX**	**MIN**	**TYPICALLY**	**MAX**
Pb	-	-	-	-	-	-	-	-	-
Cd	0.034	0.068	0.102	0.068	0.136	0.204	0.102	0.204	0.306
Zn	0.015	0.031	0.046	0.031	0.062	0.093	0.046	0.093	0.139
Cr	0.192	0.384	0.576	0.384	0.768	**1.152**	0.576	**1.152**	**1.727**
Ni	0.675	**1.350**	**2.025**	**1.350**	**2.700**	**4.050**	**2.025**	**4.050**	**6.075**
Cu	**17.563**	**35.126**	**52.689**	**35.126**	**70.252**	**105.378**	**52.689**	**105.378**	**158.067**
As	0.009	0.018	0.028	0.018	0.037	0.055	0.028	0.055	0.083
Co	-	-	-	-	-	-	-	-	-
Sb	-	-	-	-	-	-	-	-	-
Se	-	-	-	-	-	-	-	-	-
Mn	-	-	-	-	-	-	-	-	-

**Table 9:** Average LCR (Lifetime Cancer Risk) values obtained for the analyzed samples of tattoo and permanent makeup inks by type of tattoo performed.

Ink Type/Element	Contour			Realism			Filling		
**Tattoo**	**MIN**	**TYPICALLY**	**MAX**	**MIN**	**TYPICALLY**	**MAX**	**MIN**	**TYPICALLY**	**MAX**
Pb	1.70 × 10^−7^	3.41 × 10^−7^	5.11 × 10^−7^	3.41 × 10^−7^	6.82 × 10^−7^	1.02 × 10^−6^	5.11 × 10^−7^	1.02 × 10^−6^	1.53 × 10^−6^
Cd	8.38 × 10^−11^	1.68 × 10^−10^	2.51 × 10^−10^	1.68 × 10^−10^	3.35 × 10^−10^	5.03 × 10^−10^	2.51 × 10^−10^	5.03 × 10^−10^	7.54 × 10^−10^
Cr	7.64 × 10^−10^	1.53 × 10^−9^	2.29 × 10^−9^	1.53 × 10^−9^	3.06 × 10^−9^	4.59 × 10^−9^	2.29 × 10^−9^	4.59 × 10^−9^	6.88 × 10^−9^
Ni	2.29 × 10^−5^	4.59 × 10^−5^	6.88 × 10^−5^	4.59 × 10^−5^	9.17 × 10^−5^	**1.38 × 10^−4^**	6.88 × 10^−5^	**1.38 × 10^−4^**	**2.06 × 10^−4^**
As	2.17 × 10^−8^	4.33 × 10^−8^	6.50 × 10^−8^	4.33 × 10^−8^	8.66 × 10^−8^	1.30 × 10^−7^	6.50 × 10^−8^	1.30 × 10^−7^	1.95 × 10^−7^
**Makeup**									
Pb	6.31 × 10^−7^	1.26 × 10^−6^	1.89 × 10^−6^	1.26 × 10^−6^	2.52 × 10^−6^	3.78 × 10^−6^	1.89 × 10^−6^	3.78 × 10^−6^	5.68 × 10^−6^
Cd	1.67 × 10^−10^	3.35 × 10^−10^	5.02 × 10^−10^	3.35 × 10^−10^	6.69 × 10^−10^	1.00 × 10^−9^	5.02 × 10^−10^	1.00 × 10^−9^	1.51 × 10^−9^
Cr	1.12 × 10^−9^	2.23 × 10^−9^	3.35 × 10^−9^	2.23 × 10^−9^	4.47 × 10^−9^	6.70 × 10^−9^	3.35 × 10^−9^	6.70 × 10^−9^	1.01 × 10^−8^
Ni	2.47 × 10^−5^	4.94 × 10^−5^	7.41 × 10^−5^	4.94 × 10^−5^	9.88 × 10^−5^	**1.48 × 10^−4^**	7.41 × 10^−5^	**1.48 × 10^−4^**	**2.22 × 10^−4^**
**Tattoo & Makeup**									
Cd	8.94 × 10^−11^	1.79 × 10^−10^	2.68 × 10^−10^	1.79 × 10^−10^	3.58 × 10^−10^	5.36 × 10^−10^	2.68 × 10^−10^	5.36 × 10^−10^	8.05 × 10^−10^
Cr	6.40 × 10^−10^	1.28 × 10^−9^	1.92 × 10^−9^	1.28 × 10^−9^	2.56 × 10^−9^	3.84 × 10^−9^	1.92 × 10^−9^	3.84 × 10^−9^	5.76 × 10^−9^
Ni	1.27 × 10^−5^	2.54 × 10^−5^	3.81 × 10^−5^	2.54 × 10^−5^	5.08 × 10^−5^	7.62 × 10^−5^	3.81 × 10^−5^	7.62 × 10^−5^	**1.14 × 10^−4^**
As	1.56 × 10^−8^	3.12 × 10^−8^	4.68 × 10^−8^	3.12 × 10^−8^	6.24 × 10^−8^	9.36 × 10^−8^	4.68 × 10^−8^	9.36 × 10^−8^	1.40 × 10^−7^

## 4. Discussion

The European Union’s new guidelines on tattoo ink safety aim to protect public health by restricting the use of hazardous chemicals. The regulations promote the development of safer alternatives and raise consumer awareness of the risks associated with tattooing. Although the new regulations pose challenges for the tattoo industry, they also offer opportunities for innovation and improved safety standards. The popularity of body tattooing has been growing steadily for several decades, and social acceptance of this form of body decoration is also increasing. However, little is still known about the long-term health effects of tattoos. Between 2019 and 2025, few studies were conducted on the heavy metal content of tattoo and permanent makeup inks, revealing varying levels of contamination in these products; several literature reviews have been published [30,31,32,33,34,35,36,37,38]. The results of the presented study show that the “new” standards are still not being met in UE. What is more, based on the estimated exposure, the values considered “safe” are not entirely so.

The results of the present study provide a comprehensive assessment of heavy metal contamination in tattoo and permanent makeup (PMU) inks available on the European market after the implementation of REACH restrictions in 2022. Overall, our findings indicate that, despite regulatory efforts, a substantial proportion of products still contain hazardous metals at levels posing potential health risks. Both non-carcinogenic and carcinogenic risk assessments (based on SED, MoS, HQ, and LCR) highlight copper (Cu), nickel (Ni), chromium (Cr(VI)), and arsenic (As) as priority contaminants. Cu and Ni were most relevant for non-cancer effects, while Ni predominantly contributed to carcinogenic risk. The non-carcinogenic risk analysis demonstrates that the degree of exposure varies depending on the tattooing method and ink usage. Contouring generally produces lower exposure, with Cu being the most concerning element even at minimum ink consumption. Realistic shading and filling techniques, however, result in higher systemic exposure, particularly for Ni, Cr(VI), and Cu, indicating that larger or more saturated tattoos may increase the likelihood of adverse effects. Similarly, the carcinogenic risk assessment revealed that nickel consistently poses the highest cancer risk across scenarios. These results underscore the importance of rigorous quality control and monitoring to minimize long-term health hazards associated with tattooing and PMU.

In 2021, Andreou et al. [30] conclude that while the safety of tattoo and PMU colorants has improved (especially in regulated jurisdictions), significant knowledge gaps remain (e.g., long-term effects, migration, decomposition by-products). The authors advocate for more stringent regulations, better analytical methods, improved labeling, and increased oversight to ensure safer use of tattoo/PMU colorants. They emphasize that the goal is not to ban tattooing or PMU, but to make the colorants used as safe as reasonably possible by restricting hazardous substances and improving transparency.

Nickel, chromium, cobalt, and cadmium are the most potent skin sensitizers/allergens, while arsenic carries the greatest long-term carcinogenic risk. Others (lead, copper, antimony, selenium, manganese, zinc) are more often linked to irritation, discoloration, or eczema-like effects; however, systemic toxicity is also possible with chronic dermal absorption. Comparisons with previous studies indicate general consistency regarding the presence of hazardous metals. In a report by Piccinini et al. [31], it was noted that heavy metals such as nickel, chromium, cobalt, and lead were detected in tattoo and permanent makeup ink samples (in approximately 9% of the samples analyzed). The results indicated that the concentrations of these metals often exceeded European standards, which may pose a health risk to users. This report was presented in 2016 and was the basis for the introduction of more restrictive regulations currently in force in the EU. The study by Wang et al. [32], from 2021, indicated that 93% of the purchased tattoo inks did not comply with EU/CoE labeling requirements (ResAP, etc.); 50% of the inks declared at least one pigment incorrectly (i.e., the label differed from what was detected); iron, aluminium, titanium, and copper (most in green/blue inks) were the main metals detected in the inks; total chromium (0.35–139 mg/kg) and nickel (0.1–41 mg/kg) were found in almost all samples. In our own study, these two elements were also detected in a significant number of the samples analyzed—chromium concentrations above the LOQ were found in all samples, and nickel in more than half (51%). In a study conducted by Di Gaudio et al. [33], published in 2023, the authors indicated that some tattoo inks on the market exceed permissible metal limits (notably Zn and Cr in this data set). The authors suggested that the large variability between samples suggests that quality control and standardization are lacking in the tattoo ink industry. The study indicated that four out of the tested 16 samples would be considered unsafe under regulatory criteria—this underscores the importance of screening and enforcement.

In a study analyzing the heavy metal content in tattoo inks available on the Turkish market [34], it was indicated that in some inks, lead and especially chromium levels exceeded recommended limits (e.g., those set by the Council of Europe), ink exposure decreased keratinocyte viability in a dose-dependent and color-dependent manner (i.e., higher concentrations or certain ink colors produced greater cytotoxicity), IL-18 release (a proinflammatory cytokine) increased significantly in nearly all ink- or metal-exposed groups, except for one case (chromium and one black ink from brand I), where the increase was not significant. Another study conducted by Sozer Karadagli et al. [35] addresses the concern that metal contaminants in tattoo inks may pose risks for skin hypersensitivity, inflammation, or even systemic toxicity, given that inks are injected into the dermis. Some ink samples contained copper levels above allowable limits (for soluble copper)—this raises concerns about oxidative stress, cytotoxicity, or allergenic potential of copper at high exposures. A recent clinical control study conducted at Lund University in Sweden [36] has revealed a potential link between tattoos and lymphoma, a cancer of the lymphatic system. The researchers [36] analyzed all cases of malignant lymphoma diagnosed between 2007 and 2017 in people aged 20–60 listed in the Swedish National Cancer Registry. The study population consisted of 11,905 people. Even after taking into account risk factors such as smoking and age, the researchers [36] found that the likelihood of developing malignant lymphoma was 21% higher among people who had at least one tattoo. The association was strongest for diffuse large B-cell lymphoma (DLBCL) and follicular lymphoma (FL). Interestingly, according to the study results, the size of the tattoo did not affect the risk of cancer. Another intriguing finding was that laser tattoo removal resulted in an increased risk. The exact mechanisms behind this link are still unclear, but Swedish scientists believe that it is probably related to the body’s reaction to the ink. When it is injected under the skin, the body interprets it as a foreign substance, triggering inflammation and the activation of the immune system. Studies [36,37] have shown, among other things, that ink can migrate to lymph nodes. However, researchers [36] emphasize that they still do not have sufficient evidence to conclusively state that there is a link between tattoos and an increased risk of lymphoma; further detailed research is needed. Nevertheless, people with tattoos should be aware of the potential risk and consult a doctor if they experience any worrying symptoms. In comparison with the presented Turkish and Swedish data, our own findings further emphasize the potential health risks associated with tattoo and permanent makeup inks. Specifically, the cancer risk assessment conducted in this study demonstrated that such a risk does exist, largely due to the presence of contaminants such as lead and nickel in the analyzed products. These results highlight the importance of continuous monitoring and stricter control of these inks to mitigate potential long-term health consequences.

In a study published in 2018 [38], which analyzed 29 tattoo inks produced in Europe and the United States, the levels of Cr(VI) were determined; risk characterization was performed by calculating the systemic exposure dosage (SED) and margin of safety (MoS). According to the study, 90% of inks contained Cr(VI) (range: 0.2–4.1 mg/kg), i.e., above the maximum allowed level, and no information appeared on the label. More than 1 mg/kg Cr(VI) was detected in 27.6% of inks; these might represent a possible cause of dermal adverse reactions. Exposure to Cr(VI) in inks resulted in negligible SED values and MoS values of >100 (safety threshold), indicating no appreciable systemic risk. In our own study, the estimated concentration range of hexavalent chromium (Cr(VI)) in tattoo and permanent makeup inks was found to be 0.1–2.5 mg/kg. Levels exceeding the current European Union regulatory limits were identified in 16 out of 41 analyzed samples. A Margin of Safety (MoS) below 100 was only observed under the scenario of maximum product use within the group of inks intended for permanent makeup. In contrast, the Hazard Quotient (HQ) exceeded 1 across all product groups when assuming maximum usage under different exposure scenarios.

Such clear identification of the highest-priority elements should translate into intensified production and quality control of these components in order to achieve a real reduction in risks. A good practice, as shown by research conducted by Serup, Severin, and Hammershøy [39], is the legal obligation for tattoo artists in Denmark to register inks in an electronic system (InkBase), which has been in place since 2018. Local studio databases are linked to a central database, which allows the following to be recorded while maintaining personal data protection: number of customers, tattoo sessions, ink bottle numbers, brand, pigment color (CI—Color Index), and other data. Based on the data reported to this system, it was observed that between March 2018 and the end of 2019, 39,687 people visited 108 tattoo studios and underwent 50,604 tattoo sessions. During these sessions, 109,720 bottles of ink were used, of which 10,833 (less than 10%) were marked with a CI (Color Index) code, which allowed the pigment used in the ink to be identified. It was indicated that as much as 98.1% of the inks used came from the US, which, as emphasized, may hinder regulatory oversight and the identification of local or European manufacturers. The authors’ conclusions emphasize that the pre-regulatory database of actual ink use is an important starting point for analyzing how regulations will affect ink selection, customer safety, and tattoo industry practices. In the present study, all samples of inks intended for tattooing, as well as those marketed for both tattooing and permanent makeup, were manufactured in the United States, according to information provided on the manufacturers’ websites. Altogether, these accounted for 88% of the analyzed samples. Only the inks dedicated exclusively to permanent makeup originated from other countries, namely three samples from Chinese producers and two samples from a German manufacturer.

A notable strength of the present study is the relatively large group of tattoo inks analyzed, which is considerable compared to other currently available investigations. This provides a broader and more representative overview of the products used in this category. A notable and critical limitation of this study is the very limited number of products intended for permanent makeup (PMU) included in the analysis. The small sample size substantially constrains the robustness, representativeness, and generalizability of the findings, thereby limiting the extent to which definitive conclusions can be drawn regarding the safety and risk profile of PMU products. Consequently, the present results should be interpreted with considerable caution. To ensure a more comprehensive and reliable assessment of potential health risks, future research should markedly expand both the scope and the sample size of PMU products analyzed, thereby enabling a more rigorous evaluation of variability and potential hazards within this product category. An added value of this work is the health risk assessment performed for individuals receiving tattoos or permanent makeup. Compared to previous publications, this constitutes a novel contribution, as it evaluates both non-carcinogenic and carcinogenic effects, thereby underscoring the real potential health hazards that may result from exposure to heavy metals present in these products.

### 4.1. Practical Implications

These findings indicate a clear need for coordinated action to ensure the safety of tattoo and permanent makeup inks. Regulatory authorities should enhance monitoring of products placed on the market and enforce compliance with REACH restrictions, particularly those concerning hazardous metals. Manufacturers are encouraged to tighten quality assurance of pigment sourcing and formulation processes. Tattoo artists should consciously choose certified inks with transparent documentation and communicate the potential risks to clients. Consumers should be aware that tattooing introduces substances that can remain in the body long-term and should seek professional advice when selecting products. Implementing these measures collectively can significantly reduce the burden of preventable, ink-related health outcomes in the population.

### 4.2. Limitations and Strengths

This study provides one of the first comprehensive evaluations of heavy metal contamination in tattoo and PMU inks available on the European market after the implementation of the 2022 REACH restrictions. A particular strength is the dual approach combining quantitative chemical analysis of 12 regulated trace elements with a toxicological risk assessment based on dermal exposure modeling (SED, MoS, HQ, LCR). This allows for a more realistic assessment of potential health risks compared with studies focused solely on chemical composition. Additionally, the inclusion of products widely distributed within the EU increases the practical relevance of the findings for current consumer safety.

However, several limitations must be acknowledged. The sample size did not cover the entire diversity of brands, formulations, and pigments available on the European market, and thus, the results may not be fully generalizable. Only pre-mixed liquid inks were included, whereas powdered pigments for custom mixing were excluded. Chromium speciation was not analytically determined; therefore, Cr(VI) values used in the risk assessment were conservatively estimated as 1% of total chromium. The dermal absorption factors applied in modeling were drawn from available literature and may not fully reflect chronic intradermal exposure. Although these limitations introduce uncertainty, the overall consistency of exceedances and risk estimates suggests that the identified concerns are robust and warrant continued regulatory monitoring and enforcement.

## 5. Conclusions

Tattoo and permanent makeup inks represent complex chemical formulations that require strict safety controls due to the long-term presence of their components within the human body. The results of this study demonstrate that several hazardous metals found in commercially available inks may pose both chronic and carcinogenic health risks to users. These findings reinforce the need for consumers to be aware of the potential consequences of tattooing and to make informed choices regarding tattoo studios and ink products.

The outcomes of this work underscore a growing public health concern. Strengthening regulatory enforcement should become an immediate priority, particularly through systematic surveillance of inks on the market, restrictions on pigments with known toxicological profiles, and mandatory documentation of chemical composition. Manufacturers must be obligated to reduce the presence of contaminants at the source, while tattoo artists should play an active role in guiding clients toward safer, certified products.

Further research remains essential to comprehensively evaluate the health consequences of tattoo pigments. Key priorities include long-term epidemiological investigations assessing cancer incidence and other chronic outcomes among tattooed populations, in vivo absorption and toxicokinetic studies to better understand systemic exposure, and detailed assessments of metal migration and accumulation in tissues such as lymph nodes and internal organs. Expanding scientific knowledge in these areas will support evidence-based policy development and ultimately strengthen consumer protection.

## Figures and Tables

**Table 1:** List of heavy metals regulated by REACH in tattoo and permanent makeup inks [20].

Element	CAS Number	Concentration Limit (By Weight) [%]
As	7440-38-2	0.00005
Cd	7440-43-9	0.00005
Co	7440-48-4	0.00005
Cr (VI)	7440-47-3	0.00005
Cu	7440-50-8	0.025
Hg	7439-97-6	0.00005
Ni	7440-02-0	0.0005
Pb	7439-92-1	0.00007
Sb	7440-36-0	0.00005
Se	7782-49-2	0.0002
Zn	7440-66-6	0.2

**Table 2:** The wavelengths, calibration ranges, *r*^2^ values, recoveries, LOD, and LOQ of analytical techniques.

Trace Element	Analytical Technique	Wavelength [nm]	Calibration Range	*r*^2^ Value	Recovery [%]	RSD [%]	LOD [mg/kg]	LOQ [mg/kg]
Zn	ICP-OES	213.857	0.5–3.0 mg/L	0.999	104.5	1.2	3.87	7.80
Cr		205.571	0.5–3.0 mg/L	0.999	103.3	4.5	2.58	3.68
Ni		231.604	0.5–3.0 mg/L	0.999	99.2	0.7	5.48	7.40
Cu		324.754	0.5–3.0 mg/L	0.999	98.5	1.7	4.31	5.03
As		193.695	0.5–3.0 mg/L	0.999	103.6	4.9	5.98	10.95
Co		238.892	0.5–3.0 mg/L	0.999	101.0	2.4	2.46	3.30
Sb		217.581	0.5–3.0 mg/L	0.999	96.2	6.5	7.46	11.58
Se		196.026	0.5–3.0 mg/L	0.999	101.5	5.1	8.83	14.00
Mn		257.611	1.0–10.0 mg/L	0.999	95.7	0.4	2.25	2.35
Cd	ET-AAS	228.8	5.0–20.0 µg/L	0.997	97.2	<20	0.04	0.08
Pb		217.0	10.0–40.0 µg/L	0.999	104.0	<20	0.44	0.80
Hg	CV-AFS	254.0	0.0–20.0 µg/L	0.997	99.7	-	0.004	0.008

**Table 3:** Measurement conditions and calibration parameters of analytical techniques.

Parameter	ICP-OES	ET-AAS	CV-AFS
Calibration type	maximum	least squares	least squares
Calibration of failure criteria: *r*^2^	≥0.995	≥0.995	≥0.995
RSD for standards	<10%	<10%	<10%
Gas flow Ar 99.999% [L/min]	12–13	3	2.50
Generator power [W]	1000	-	-
Nebulizer	teflon	-	-
Peristaltic pump speed	15 [rpm]	-	100 [%]
Ambient temperature of the equipment	18–24 ± 2 °C	10–35 °C	3–30 °C
Air humidity in the equipment’s surroundings	20–80%	3–30%	<80%

**Table 4:** Estimated tattoo ink consumption for three skin surfaces in different tattooing styles.

Skin Surface [cm^2^]	Style	Min [g]	Typical [g]	Max [g]
5	contour	0.05	0.10	0.15
	realism	0.10	0.20	0.30
	filling	0.15	0.25	0.40
10	contour	0.10	0.20	0.30
	realism	0.20	0.40	0.60
	filling	0.30	0.50	0.80
100	contour	1.00	2.00	3.00
	realism	2.00	4.00	6.00
	filling	3.00	5.00	8.00

**Table 5:** Descriptive statistics of the distribution of concentrations of analyzed elements in all samples of inks.

Parameter	Element Concentration [mg/kg]										
	Pb	Cd	Zn	Cr	Ni	Cu	As	Co	Sb	Se	Mn
MIN	0.87	0.09	8.20	7.25	7.82	9.50	11.22	3.56	12.22	14.33	4.38
Q1	1.19	0.09	10.76	11.91	13.16	14.94	15.68	22.13	12.62	17.08	6.48
MEDIAN	2.00	0.09	20.07	18.95	34.15	36.79	18.36	54.44	15.25	19.83	23.76
Q3	2.18	0.10	33.29	106.65	115.89	404.36	22.07	62.38	18.82	20.57	108.50
MEAN	2.81	0.13	38.81	58.97	62.98	1751.01	20.06	44.14	16.03	18.49	169.13
MAX	7.83	0.34	202.18	246.39	207.33	25,700.80	53.57	76.00	21.26	21.31	1089.81
SD	2.86	0.08	54.94	63.02	58.72	4676.02	9.01	29.22	3.71	3.68	353.22
VARIANCE	8.15	6.39 × 10^−3^	3.02 × 10^3^	3.97 × 10^3^	3.97 × 10^−1^	3.45 × 10^3^	2.19 × 10^7^	8.12 × 10^1^	8.54 × 10^2^	1.38 × 10^1^	1.35 × 10^1^

**Table 6:** Number of ink samples for which the calculated parameter exceeded the permissible values.

Parameter	Tattoo Method	MIN											TYPICAL											MAX										
		Pb	Cd	Zn	Cr	Ni	Cu	As	Co	Sb	Se	Mn	Pb	Cd	Zn	Cr	Ni	Cu	As	Co	Sb	Se	Mn	Pb	Cd	Zn	Cr	Ni	Cu	As	Co	Sb	Se	Mn
MoS < 100	Contour	0	1	6	0	1	31	1	0	8	2	7	0	2	13	8	7	35	2	4	8	3	7	0	6	18	12	10	35	13	4	8	3	7
	Realism	0	2	13	7	8	35	2	4	8	3	9	0	10	20	15	11	35	17	4	8	3	9	1	10	20	18	15	35	20	4	8	3	9
	Filling	0	6	18	12	10	35	13	4	8	3	9	1	10	20	18	15	35	20	4	8	3	9	1	10	20	18	17	35	20	5	8	3	9
HQ > 1	Contour	0	0	0	0	10	11	0	0	8	3	6	0	0	0	9	14	11	0	4	8	3	9	0	0	0	12	17	1	0	4	8	3	9
	Realism	0	0	0	7	15	11	0	4	8	3	9	0	0	0	15	18	22	0	4	8	3	9	1	0	2	18	21	25	0	4	8	3	9
	Filling	0	0	0	12	17	15	0	4	8	3	9	1	0	2	18	19	25	0	4	8	3	9	1	1	2	18	24	28	0	5	8	3	9
LCR > 1 × 10^−4^	Contour	0	0	0	0	0	0	0	0	0	0	0	0	0	0	0	2	0	0	0	0	0	0	0	0	0	0	8	0	0	0	0	0	0
	Realism	0	0	0	0	2	0	0	0	0	0	0	0	0	0	0	8	0	0	0	0	0	0	0	0	0	0	11	0	0	0	0	0	0
	Filling	0	0	0	0	8	0	0	0	0	0	0	0	0	0	0	11	0	0	0	0	0	0	0	0	0	0	12	0	0	0	0	0	0

**Table 7:** Average MoS (Margin of Safety) values obtained for the analyzed samples of tattoo and permanent makeup inks by type of tattoo performed.

**Ink Type/Element**	**Contour**			**Realism**			**Filling**		
**Tattoo**	**MIN**	**TYPICALLY**	**MAX**	**MIN**	**TYPICALLY**	**MAX**	**MIN**	**TYPICALLY**	**MAX**
Pb	2793.35	1396.67	931.12	1396.67	698.34	465.56	931.12	465.56	310.37
Cd	314.42	157.21	104.81	157.21	**78.60**	**52.40**	104.81	**52.40**	**34.94**
Zn	151.25	**75.62**	**50.42**	**75.62**	**37.81**	**25.21**	**50.42**	**25.21**	**16.81**
Cr	1461.69	730.85	487.23	730.85	365.42	243.62	487.23	243.62	162.41
Ni	864.05	432.03	288.02	432.03	216.01	144.01	288.02	144.01	**96.01**
Cu	**34.98**	**17.49**	**11.66**	**17.49**	**8.74**	**5.83**	**11.66**	**5.83**	**3.89**
As	261.09	130.55	**87.03**	130.55	**65.27**	**43.52**	**87.03**	**43.52**	**29.01**
Co	692.76	346.38	230.92	346.38	173.19	115.46	230.92	115.46	76.97
Sb	**0.73**	**0.37**	**0.24**	**0.37**	**0.18**	**0.12**	**0.24**	**0.12**	**0.08**
Se	**0.05**	**0.02**	**0.02**	**0.02**	**0.01**	**0.01**	**0.02**	**0.01**	**0.01**
Mn	**0.71**	**0.36**	**0.24**	**0.36**	**0.18**	**0.12**	**0.24**	**0.12**	**0.08**
**Ink type/Element**	**Contour**			**Realism**			**Filling**		
**Makeup**	**MIN**	**TYPICALLY**	**MAX**	**MIN**	**TYPICALLY**	**MAX**	**MIN**	**TYPICALLY**	**MAX**
Pb	985.11	492.55	328.37	492.55	246.28	164.18	328.37	164.18	109.46
Cd	205.87	102.94	**68.62**	102.94	**51.47**	**34.31**	**68.62**	**34.31**	**22.87**
Zn	**79.38**	**39.69**	**26.46**	**39.69**	**19.85**	**13.23**	**26.46**	**13.23**	**8.82**
Cr	638.41	319.21	212.80	319.21	159.60	106.40	212.80	106.40	**70.93**
Ni	599.75	299.88	199.92	299.88	149.94	**99.96**	199.92	**99.96**	**66.64**
Cu	**90.22**	**45.11**	**30.07**	**45.11**	**22.56**	**15.04**	**30.07**	**15.04**	**10.02**
As	-	-	-	-	-	-	-	-	**-**
Co	397.91	198.95	132.64	198.95	**99.48**	**66.32**	**132.64**	**66.32**	**44.21**
Sb	-	-	-	-	-	-	-	-	**-**
Se	-	-	-	-	-	-	-	-	**-**
Mn	**0.61**	**0.31**	**0.20**	**0.31**	**0.15**	**0.10**	**0.20**	**0.10**	**0.07**
**Ink type/Element**	**Contour**			**Realism**			**Filling**		
**Tattoo & Makeup**	**MIN**	**TYPICALLY**	**MAX**	**MIN**	**TYPICALLY**	**MAX**	**MIN**	**TYPICALLY**	**MAX**
Pb	-	-	-	-	-	-	-	-	-
Cd	295.51	147.75	98.50	147.75	**73.88**	**49.25**	**98.50**	**49.25**	**32.83**
Zn	241.91	120.96	80.64	120.96	**60.48**	**40.32**	**80.64**	**40.32**	**26.88**
Cr	1166.55	583.28	388.85	583.28	291.64	194.43	388.85	194.43	129.62
Ni	519.47	259.74	173.16	259.74	129.87	86.58	173.16	**86.58**	**57.72**
Cu	**29.44**	**14.72**	**9.81**	**14.72**	**7.36**	**4.91**	**9.81**	**4.91**	**3.27**
As	347.81	173.91	115.94	173.91	**86.95**	**57.97**	115.94	**57.97**	**38.65**
Co	-	-	-	-	-	-	-	-	-
Sb	-	-	-	-	-	-	-	-	-
Se	-	-	-	-	-	-	-	-	-
Mn	-	-	-	-	-	-	-	-	-

## Data Availability

The original contributions presented in this study are included in the article/Supplementary Materials. Further inquiries can be directed to the corresponding author.

## Supplementary Materials

The following supporting information can be downloaded at: https://www.mdpi.com/article/10.3390/toxics13110934/s1, Table S1: Brands of tattoo inks analyzed in the study; Table S2: Concentration [mg/kg] of selected elements in analyzed samples of tattoo and permanent makeup inks; Table S3: Systemic exposure dose (SED) [mg/kg body weight/day] calculated for the analyzed elements contained in tattoo inks used to create a 5 cm tattoo using the contouring method; Table S4: Systemic exposure dose (SED) [mg/kg body weight/day] calculated for the analyzed elements contained in tattoo inks used to create a 5 cm tattoo using the realism method; Table S5: Systemic exposure dose (SED) [mg/kg body weight/day] calculated for the analyzed elements contained in tattoo inks used to create a 5 cm tattoo using the filling method; Table S6: Margin of Safety (MoS) calculated for the analyzed elements contained in tattoo inks used to create a 5 cm tattoo using the contour method; Table S7: Margin of Safety (MoS) calculated for the analyzed elements contained in tattoo inks used to create a 5 cm tattoo using the realism method; Table S8: Margin of Safety (MoS) calculated for the analyzed elements contained in tattoo inks used to create a 5 cm tattoo using the filling method; Table S9: Hazard Quotient (HQ) calculated for the analyzed elements contained in tattoo inks used to create a 5 cm tattoo using the contour method; Table S10: Hazard Quotient (HQ) calculated for the analyzed elements contained in tattoo inks used to create a 5 cm tattoo using the realism method; Table S11: Hazard Quotient (HQ) calculated for the analyzed elements contained in tattoo inks used to create a 5 cm tattoo using the filling method; Table S12: Lifetime Cancer Risk (LCR) calculated for the analyzed elements contained in tattoo inks used to create a 5 cm tattoo using the contour method; Table S13: Lifetime Cancer Risk (LCR) calculated for the analyzed elements contained in tattoo inks used to create a 5 cm tattoo using the realism method; Table S14: Lifetime Cancer Risk (LCR) calculated for the analyzed elements contained in tattoo inks used to create a 5 cm tattoo using the filling method.

## Abbreviations

The following abbreviations are used in this manuscript:

AA	Amount Applied
BW	Body Weight
C	Concentration
CAS number	Chemical Abstracts Service Registry Number
CI	Color Index
CSF	Cancer Slope Factor
DAF	Dermal Absorption Factor
EU	European Union
HQ	Hazard Quotient
LCR	Lifetime Cancer Risk
LOQ	Limit of Quantification
MF	Modifying Factor
MoS	Margin of Safety
NOAEL	No Observed Adverse Effect Level
REACH	Registration, Evaluation, Authorization, and Restriction of Chemicals
RF	Retention Factor
RfD	Reference Dose
SED	Systemic Exposure Dose
SSA	Skin Surface Area
UF	Uncertainty Factor
