# Supplementary material for: Heavy Metal Content in Tattoo and Permanent Makeup Inks and European Standards—Is There Still a Health Risk?
Source: Toxics. 2025 Oct 30;13(11):934. doi: 10.3390/toxics13110934 (PMC12656104; doi:10.3390/toxics13110934)
Supplement: Supplementary file 1 [file toxics-13-00934-s001.zip › Supplementary materials.pdf]

Table S1 Brands of tattoo inks analyzed in the study.

Brand name	Country of manufacture	Brand coding used in the manuscript (text, tables)
Eternal Ink	USA	1
Intenze	USA	2
Radiant	USA	3
Goochie	China	4
PM Permanent Make-up	China	5
Hanami	Germany	6
World Famous Tattoo Ink	USA	7

Table S2 Concentration [mg/kg] of selected elements in analyzed samples of tattoo and permanent makeup inks.

No	Product purpose	Color	Brand	Comments	Pb	Cd	Zn	Cr	Ni	Cu	As	Co	Sb	Se	Mn
1	**TATTOO INK**	white	1	**	-	-	-	18.95	7.82	41.01	27.71	-	14.31	-	-
2		brown		**	-	-	31.14	111.19	29.21	22.33	22.10	76.00	21.26	19.83	23.76
3		black			-	0.09	-	23.26	14.17	-	-	-	-	-	-
4		red			-	-	-	7.25	-	-	-	-	-	-	-
5		purple			-	-	-	14.23	8.60	547.19	16.22	-	-	-	-
6		blue			-	-	12.59	11.22	-	25700.80	-	-	-	-	-
7		orange		**	-	-	-	17.11	10.05	33.30	12.43	-	-	-	-
8		pink		**	-	-	-	14.68	-	36.79	23.67	-	12.22	-	-
9		green			-	-	-	13.25	10.12	6438.10	14.05	-	-	-	-
10		yellow			-	-	-	7.33	-	-	-	-	-	-	-
11		brown	2		1.19	-	57.00	162.62	38.05	261.52	18.14	49.93	16.19	14.33	108.50
12		black			-	-	19.71	175.97	124.03	9.50	-	-	-	-	4.83
13		red			-	-	-	18.24	-	225.78	-	-	-	-	-
14		purple			-	-	15.32	128.47	121.63	37.55	-	-	-	-	-
15		blue			-	-	-	11.21	-	3442.50	20.43	-	12.55	-	-
16		pink			-	-	-	11.91	-	35.27	23.34	-	12.65	-	-
17		green			-	-	-	9.46	-	5501.61	-	-	-	-	-
18		yellow			2.00	-	20.43	191.84	113.98	11.99	-	-	-	-	6.48
19		orange			-	-	-	8.87	-	12.68	-	-	-	-	-
20		white	3		-	-	8.23	11.77	-	42.69	53.57	-	18.13	-	-
21		black			-	-	23.50	136.75	124.25	10.16	-	-	-	-	-
22		purple			-	-	-	10.75	-	43.16	17.16	-	-	-	-
23		blue			-	0.09	-	19.09	-	5539.22	16.67	-	-	-	-
24		orange			0.87	-	11.24	246.39	136.08	13.20	-	3.56	-	-	9.03
25		pink			-	-	9.29	89.18	207.33	37.21	18.58	-	-	-	-
26		green			-	0.09	-	11.31	-	1335.00	17.80	-	-	-	-
27		yellow			-	-	186.21	77.60	9.88	24.21	21.58	63.51	20.89	21.31	44.21
28	**PERMANENT MAKEUP INK**	chocolate	4		2.18	-	202.18	48.13	30.24	37.26	-	58.96	-	-	1089.81
29		black			7.83	0.34	21.91	106.65	58.27	11.73	-	-	-	-	
30		chocolate-brown	5		-	0.18	39.72	12.42	8.89	9.52	-	12.86	-	-	231.15

31		black coffee	6	-	0.09	16.25	146.33	101.02	11.71	-	-	-	-	4.38
32		brown-black		-	0.10	49.45	108.82	168.87	9.98	-	-	-	-	
33		orange		-	-	-	71.39	23.50	-	-	-	-	-	-
34		white		-	-	-	11.99	-	16.67	11.22	-	-	-	-
35		yellow		-	-	-	79.95	30.10	35.57	22.06	-	-	-	-
36		red		-	0.09	-	13.37	-	-	-	-	-	-	-
37	**TATTOO & PERMANENT MAKEUP INK**	black	7	-	-	-	49.45	50.13	-	-	-	-	-	-
38		purple		-	0.10	8.20	8.86	-	7485.44	-	-	-	-	-
39		blue		-	-	8.61	59.22	23.41	4206.31	18.76	-	-	-	-
40		brown		-	0.10	25.95	13.50	-	26.30	13.90	-	-	-	-
41		pink		-	-	9.31	127.58	61.87	32.03	11.71	-	-	-	-
Concentration limits [mg/kg]				0.7	0.5	2000.0	0.5***	5.0	250.0	0.5	0.5	0.5	2.0	-

** Information provided by the manufacturer on the ink packaging – „May contain trace amounts of Nickel”.

*** Concentration limit [mg/kg] for chromium (VI).

Table S3 Systemic exposure dose (SED) [mg/kg body weight/day] calculated for the analyzed elements contained in tattoo inks used to create a 5 cm tattoo using the contouring method.

No	Product purpose	Color	Brand	Comment	5 cm – Contour MIN											5 cm – Contour TYPICALLY											5 cm – Contour MAX										
					Pb	Cd	Zn	Cr	Ni	Cu	As	Co	Sb	Se	Mn	Pb	Cd	Zn	Cr	Ni	Cu	As	Co	Sb	Se	Mn	Pb	Cd	Zn	Cr	Ni	Cu	As	Co	Sb	Se	Mn
1	tattoo ink	white	1	**				6.77E-08	1.12E-04	1.46E-05	2.97E-05		5.11E-04						1.35E-07	2.23E-04	2.93E-05	5.94E-05		1.02E-03						2.03E-07	3.35E-04	4.39E-05	8.91E-05		1.53E-03		
2		brown		**			2.22E-04	3.97E-07	4.17E-04	7.98E-06	2.37E-05	2.17E-02	7.59E-04	3.54E-03	4.24E-03			4.45E-04	7.94E-07	8.35E-04	1.60E-05	4.73E-05	4.34E-02	1.52E-03	7.08E-03	8.49E-03			6.67E-04	1.19E-06	1.25E-03	2.39E-05	7.10E-05	6.51E-02	2.28E-03	1.06E-02	1.27E-02
3		black				3.10E-08		8.31E-08	2.02E-04								6.20E-08		1.66E-07	4.05E-04								9.31E-08		2.49E-07	6.07E-04						
4		red						2.59E-08											5.18E-08											7.76E-08							
5		purple						5.08E-08	1.23E-04	1.95E-04	1.74E-05								1.02E-07	2.46E-04	3.91E-04	3.48E-05								1.53E-07	3.69E-04	5.86E-04	5.22E-05				
6		blue					8.99E-05	4.01E-08		9.18E-03								1.80E-04	8.02E-08		1.84E-02								2.70E-04	1.20E-07		2.75E-02					
7		orange		**				6.11E-08	1.44E-04	1.19E-05	1.33E-05								1.22E-07	2.87E-04	2.38E-05	2.66E-05								1.83E-07	4.31E-04	3.57E-05	3.99E-05				
8		pink		**				5.24E-08		1.31E-05	2.54E-05		4.37E-04						1.05E-07		2.63E-05	5.07E-05		8.73E-04						1.57E-07		3.94E-05	7.61E-05		1.31E-03		

17	16	15	14	13	12	11	10	9
green	pink	blue	purple	red	black	brown	yellow	green
			2					
						4.24E-07		
			1.09E-04		1.41E-04	4.07E-04		
3.38E-08	4.25E-08	4.00E-08	4.59E-07	6.51E-08	6.28E-07	5.81E-07	2.62E-08	4.73E-08
			1.74E-03		1.77E-03	5.44E-04		1.45E-04
1.96E-03	1.26E-05	1.23E-03	1.34E-05	8.06E-05	3.39E-06	9.34E-05		2.30E-03
	2.50E-05	2.19E-05				1.94E-05		1.51E-05
						1.43E-02		
	4.52E-04	4.48E-04				5.78E-04		
						2.56E-03		
					8.63E-04	1.94E-02		
						8.48E-07		
			2.19E-04		2.82E-04	8.14E-04		
6.76E-08	8.50E-08	8.00E-08	9.18E-07	1.30E-07	1.26E-06	1.16E-06	5.23E-08	9.47E-08
			3.48E-03		3.54E-03	1.09E-03		2.89E-04
3.93E-03	2.52E-05	2.46E-03	2.68E-05	1.61E-04	6.79E-06	1.87E-04		4.60E-03
	5.00E-05	4.38E-05				3.89E-05		3.01E-05
						2.85E-02		
	9.03E-04	8.96E-04				1.16E-03		
						5.12E-03		
					1.73E-03	3.88E-02		
						1.27E-06		
			3.28E-04		4.22E-04	1.22E-03		
1.01E-07	1.28E-07	1.20E-07	1.38E-06	1.95E-07	1.89E-06	1.74E-06	7.85E-08	1.42E-07
			5.21E-03		5.32E-03	1.63E-03		4.34E-04
5.89E-03	3.78E-05	3.69E-03	4.02E-05	2.42E-04	1.02E-05	2.80E-04		6.90E-03
	7.50E-05	6.57E-05				5.83E-05		4.52E-05
						4.28E-02		
	1.35E-03	1.34E-03				1.73E-03		
						7.68E-03		
					2.59E-03	5.81E-02		

26	25	24	23	22	21	20	19	18
green	pink	orange	blue	purple	black	white	orange	yellow
			3					
		3.10E-07						7.15E-07
3.30E-08			3.14E-08					
	6.63E-05	8.03E-05			1.68E-04	5.88E-05		1.46E-04
4.04E-08	3.19E-07	8.80E-07	6.82E-08	3.84E-08	4.88E-07	4.20E-08	3.17E-08	6.85E-07
	2.96E-03	1.94E-03			1.78E-03			1.63E-03
4.77E-04	1.33E-05	4.71E-06	1.98E-03	1.54E-05	3.63E-06	1.52E-05	4.53E-06	4.28E-06
1.91E-05	1.99E-05		1.79E-05	1.84E-05		5.74E-05		
		1.02E-03						
						6.48E-04		
		1.61E-03						1.16E-03
		6.20E-07						1.43E-06
6.61E-08			6.29E-08					
	1.33E-04	1.61E-04			3.36E-04	1.18E-04		2.92E-04
8.08E-08	6.37E-07	1.76E-06	1.36E-07	7.68E-08	9.77E-07	8.41E-08	6.34E-08	1.37E-06
	5.92E-03	3.89E-03			3.55E-03			3.26E-03
9.54E-04	2.66E-05	9.43E-06	3.96E-03	3.08E-05	7.25E-06	3.05E-05	9.06E-06	8.56E-06
3.81E-05	3.98E-05		3.57E-05	3.68E-05		1.15E-04		
		2.03E-03						
						1.30E-03		
		3.23E-03						2.31E-03
		9.30E-07						2.15E-06
9.91E-08			9.43E-08					
	1.99E-04	2.41E-04			5.04E-04	1.76E-04		4.38E-04
1.21E-07	9.56E-07	2.64E-06	2.05E-07	1.15E-07	1.47E-06	1.26E-07	9.50E-08	2.06E-06
	8.89E-03	5.83E-03			5.33E-03			4.88E-03
1.43E-03	3.99E-05	1.41E-05	5.93E-03	4.62E-05	1.09E-05	4.57E-05	1.36E-05	1.28E-05
5.72E-05	5.97E-05		5.36E-05	5.52E-05		1.72E-04		
		3.05E-03						
						1.94E-03		
		4.84E-03						3.47E-03

35	34	33	32	31	30	29	28	27
**tattoo & permanent makeup ink**			**permanent makeup ink**					
yellow	white	orange	brown-black	black coffee	chocolate-brown	black	chocolate	yellow
	**7**		**6**		**5**	**4**		
						2.80E-06	7.79E-07	
			3.48E-08	3.34E-08	6.49E-08	1.21E-07		
			3.53E-04	1.16E-04	2.84E-04	1.57E-04	1.44E-03	1.33E-03
2.86E-07	4.28E-08	2.55E-07	3.89E-07	5.23E-07	4.44E-08	3.81E-07	1.72E-07	2.77E-07
4.30E-04		3.36E-04	2.41E-03	1.44E-03	1.27E-04	8.32E-04	4.32E-04	1.41E-04
1.27E-05	5.95E-06		3.57E-06	4.18E-06	3.40E-06	4.19E-06	1.33E-05	8.65E-06
2.36E-05	1.20E-05							2.31E-05
					3.67E-03		1.68E-02	1.81E-02
								7.46E-04
								3.81E-03
				7.82E-04	4.13E-02		1.95E-01	7.89E-03
						5.59E-06	1.56E-06	
			6.95E-08	6.69E-08	1.30E-07	2.42E-07		
			7.06E-04	2.32E-04	5.67E-04	3.13E-04	2.89E-03	2.66E-03
5.71E-07	8.56E-08	5.10E-07	7.77E-07	1.05E-06	8.87E-08	7.62E-07	3.44E-07	5.54E-07
8.60E-04		6.71E-04	4.82E-03	2.89E-03	2.54E-04	1.66E-03	8.64E-04	2.82E-04
2.54E-05	1.19E-05		7.13E-06	8.36E-06	6.80E-06	8.38E-06	2.66E-05	1.73E-05
4.73E-05	2.41E-05							4.63E-05
					7.35E-03		3.37E-02	3.63E-02
								1.49E-03
								7.61E-03
				1.56E-03	8.26E-02		3.89E-01	1.58E-02
						8.39E-06	2.34E-06	
			1.04E-07	1.00E-07	1.95E-07	3.64E-07		
			1.06E-03	3.48E-04	8.51E-04	4.70E-04	4.33E-03	3.99E-03
8.57E-07	1.28E-07	7.65E-07	1.17E-06	1.57E-06	1.33E-07	1.14E-06	5.16E-07	8.31E-07
1.29E-03		1.01E-03	7.24E-03	4.33E-03	3.81E-04	2.50E-03	1.30E-03	4.23E-04
3.81E-05	1.79E-05		1.07E-05	1.25E-05	1.02E-05	1.26E-05	3.99E-05	2.59E-05
7.09E-05	3.61E-05							6.94E-05
					1.10E-02		5.05E-02	5.44E-02
								2.24E-03
								1.14E-02
				2.35E-03	1.24E-01		5.84E-01	2.37E-02

**36**	red	3.13E-08		4.77E-08				6.27E-08		9.55E-08				9.40E-08		1.43E-07			
**37**	black			1.77E-07	7.16E-04					3.53E-07	1.43E-03					5.30E-07	2.15E-03		
**38**	purple	3.40E-08	5.86E-05	3.16E-08		2.67E-03		6.80E-08	1.17E-04	6.33E-08		5.35E-03		1.02E-07	1.76E-04	9.49E-08		8.02E-03	
**39**	blue		6.15E-05	2.12E-07	3.34E-04	1.50E-03	2.01E-05		1.23E-04	4.23E-07	6.69E-04	3.00E-03	4.02E-05		1.84E-04	6.35E-07	1.00E-03	4.51E-03	6.03E-05
**40**	brown	3.66E-08	1.85E-04	4.82E-08		9.39E-06	1.49E-05	7.32E-08	3.71E-04	9.64E-08		1.88E-05	2.98E-05	1.10E-07	5.56E-04	1.45E-07		2.82E-05	4.47E-05
**41**	pink		6.65E-05	4.56E-07	8.84E-04	1.14E-05	1.25E-05		1.33E-04	9.11E-07	1.77E-03	2.29E-05	2.51E-05		2.00E-04	1.37E-06	2.65E-03	3.43E-05	3.76E-05

** Information provided by the manufacturer on the ink packaging – „May contain trace amounts of Nickel”.

Table S4 Systemic exposure dose (SED) [mg/kg body weight/day] calculated for the analyzed elements contained in tattoo inks used to create a 5 cm tattoo using the realism method.

					5 cm – Realism MIN											5 cm – Realism TYPICALLY											5 cm – Realism MAX										
**No**	**Product purpose**	**Color**	**Brand**	**Comment**	**Pb**	**Cd**	**Zn**	**Cr**	**Ni**	**Cu**	**As**	**Co**	**Sb**	**Se**	**Mn**	**Pb**	**Cd**	**Zn**	**Cr**	**Ni**	**Cu**	**As**	**Co**	**Sb**	**Se**	**Mn**	**Pb**	**Cd**	**Zn**	**Cr**	**Ni**	**Cu**	**As**	**Co**	**Sb**	**Se**	**Mn**
**1**	**tattoo ink**	white	**1**	**				1.35E-07	2.23E-04	2.93E-05	5.94E-05		1.02E-03						2.71E-07	4.47E-04	5.86E-05	1.19E-04		2.04E-03						4.06E-07	6.70E-04	8.79E-05	1.78E-04		3.07E-03		
**2**		brown		**			4.45E-04	7.94E-07	8.35E-04	1.60E-05	4.73E-05	4.34E-02	1.52E-03	7.08E-03	8.49E-03			8.90E-04	1.59E-06	1.67E-03	3.19E-05	9.47E-05	8.69E-02	3.04E-03	1.42E-02	1.70E-02			1.33E-03	2.38E-06	2.50E-03	4.79E-05	1.42E-04	1.30E-01	4.56E-03	2.13E-02	2.55E-02
**3**		black				6.20E-08		1.66E-07	4.05E-04								1.24E-07		3.32E-07	8.10E-04								1.86E-07		4.98E-07	1.21E-03						
**4**		red						5.18E-08											1.04E-07											1.55E-07							
**5**		purple						1.02E-07	2.46E-04	3.91E-04	3.48E-05								2.03E-07	4.92E-04	7.82E-04	6.95E-05								3.05E-07	7.38E-04	1.17E-03	1.04E-04				
**6**		blue					1.80E-04	8.02E-08		1.84E-02	0.00E+00							3.60E-04	1.60E-07		3.67E-02								5.39E-04	2.41E-07		5.51E-02					
**7**		orange		**				1.22E-07	2.87E-04	2.38E-05	2.66E-05								2.44E-07	5.74E-04	4.76E-05	5.33E-05								3.67E-07	8.61E-04	7.14E-05	7.99E-05				
**8**		pink		**				1.05E-07		2.63E-05	5.07E-05		8.73E-04						2.10E-07		5.26E-05	1.01E-04		1.75E-03						3.15E-07		7.88E-05	1.52E-04	0.00E+00	2.62E-03		

**17**	**16**	**15**	**14**	**13**	**12**	**11**	**10**	**9**
green	pink	blue	purple	red	black	brown	yellow	green
			**2**					
						8.48E-07		
			2.19E-04		2.82E-04	8.14E-04		
6.76E-08	8.50E-08	8.00E-08	9.18E-07	1.30E-07	1.26E-06	1.16E-06	5.23E-08	9.47E-08
			3.48E-03		3.54E-03	1.09E-03		2.89E-04
3.93E-03	2.52E-05	2.46E-03	2.68E-05	1.61E-04	6.79E-06	1.87E-04		4.60E-03
	5.00E-05	4.38E-05				3.89E-05		3.01E-05
						2.85E-02		
	9.03E-04	8.96E-04				1.16E-03		
						5.12E-03		
					1.73E-03	3.88E-02		
						1.70E-06		
			4.38E-04		5.63E-04	1.63E-03		
1.35E-07	1.70E-07	1.60E-07	1.84E-06	2.61E-07	2.51E-06	2.32E-06	1.05E-07	1.89E-07
			6.95E-03		7.09E-03	2.17E-03		5.78E-04
7.86E-03	5.04E-05	4.92E-03	5.36E-05	3.23E-04	1.36E-05	3.74E-04		9.20E-03
	1.00E-04	8.75E-05				7.78E-05		6.02E-05
						5.71E-02		
	1.81E-03	1.79E-03				2.31E-03		
						1.02E-02		
					3.45E-03	7.75E-02		
						2.54E-06		
			6.57E-04		8.45E-04	2.44E-03		
2.03E-07	2.55E-07	2.40E-07	2.75E-06	3.91E-07	3.77E-06	3.48E-06	1.57E-07	2.84E-07
			1.04E-02		1.06E-02	3.26E-03		8.68E-04
1.18E-02	7.56E-05	7.38E-03	8.05E-05	4.84E-04	2.04E-05	5.60E-04		1.38E-02
	1.50E-04	1.31E-04				1.17E-04		9.03E-05
						8.56E-02		
	2.71E-03	2.69E-03				3.47E-03		
						1.54E-02		
					5.18E-03	1.16E-01		

26	25	24	23	22	21	20	19	18
green	pink	orange	blue	purple	black	white	orange	yellow
			3					
		6.20E-07						1.43E-06
6.61E-08			6.29E-08					
	1.33E-04	1.61E-04			3.36E-04	1.18E-04		2.92E-04
8.08E-08	6.37E-07	1.76E-06	1.36E-07	7.68E-08	9.77E-07	8.41E-08	6.34E-08	1.37E-06
	5.92E-03	3.89E-03			3.55E-03			3.26E-03
9.54E-04	2.66E-05	9.43E-06	3.96E-03	3.08E-05	7.25E-06	3.05E-05	9.06E-06	8.56E-06
3.81E-05	3.98E-05		3.57E-05	3.68E-05		1.15E-04		
		2.03E-03						
						1.30E-03		
		3.23E-03						2.31E-03
		1.24E-06						2.86E-06
1.32E-07			1.26E-07					
	2.65E-04	3.21E-04			6.71E-04	2.35E-04		5.84E-04
1.62E-07	1.27E-06	3.52E-06	2.73E-07	1.54E-07	1.95E-06	1.68E-07	1.27E-07	2.74E-06
	1.18E-02	7.78E-03			7.10E-03			6.51E-03
1.91E-03	5.32E-05	1.89E-05	7.91E-03	6.17E-05	1.45E-05	6.10E-05	1.81E-05	1.71E-05
7.63E-05	7.96E-05		7.14E-05	7.35E-05		2.30E-04		
		4.07E-03						
						2.59E-03		
		6.45E-03						4.63E-03
		1.86E-06						4.29E-06
1.98E-07			1.89E-07					
	3.98E-04	4.82E-04			1.01E-03	3.53E-04		8.76E-04
2.42E-07	1.91E-06	5.28E-06	4.09E-07	2.30E-07	2.93E-06	2.52E-07	1.90E-07	4.11E-06
	1.78E-02	1.17E-02			1.07E-02			9.77E-03
2.86E-03	7.97E-05	2.83E-05	1.19E-02	9.25E-05	2.18E-05	9.15E-05	2.72E-05	2.57E-05
1.14E-04	1.19E-04	0.00E+00	1.07E-04	1.10E-04		3.44E-04		
		6.10E-03						
						3.89E-03		
		9.68E-03						6.94E-03

35	34	33	32	31	30	29	28	27
tattoo & permanent makeup ink			permanent makeup ink					
yellow	white	orange	brown-black	black coffee	chocolate-brown	black	chocolate	yellow
	7		6		5	4		
						5.59E-06	1.56E-06	
		0.00E+00	6.95E-08	6.69E-08	1.30E-07	2.42E-07		
			7.06E-04	2.32E-04	5.67E-04	3.13E-04	2.89E-03	2.66E-03
5.71E-07	8.56E-08	5.10E-07	7.77E-07	1.05E-06	8.87E-08	7.62E-07	3.44E-07	5.54E-07
8.60E-04		6.71E-04	4.82E-03	2.89E-03	2.54E-04	1.66E-03	8.64E-04	2.82E-04
2.54E-05	1.19E-05		7.13E-06	8.36E-06	6.80E-06	8.38E-06	2.66E-05	1.73E-05
4.73E-05	2.41E-05							4.63E-05
					7.35E-03		3.37E-02	3.63E-02
								1.49E-03
								7.61E-03
				1.56E-03	8.26E-02		3.89E-01	1.58E-02
						1.12E-05	3.12E-06	
			1.39E-07	1.34E-07	2.59E-07	4.85E-07		
		0.00E+00	1.41E-03	4.64E-04	1.13E-03	6.26E-04	5.78E-03	5.32E-03
1.14E-06	1.71E-07	1.02E-06	1.55E-06	2.09E-06	1.77E-07	1.52E-06	6.88E-07	1.11E-06
1.72E-03		1.34E-03	9.65E-03	5.77E-03	5.08E-04	3.33E-03	1.73E-03	5.64E-04
5.08E-05	2.38E-05		1.43E-05	1.67E-05	1.36E-05	1.68E-05	5.32E-05	3.46E-05
9.46E-05	4.81E-05							9.25E-05
					1.47E-02		6.74E-02	7.26E-02
								2.98E-03
								1.52E-02
			0.00E+00	3.13E-03	1.65E-01	0.00E+00	7.78E-01	3.16E-02
						1.68E-05	4.67E-06	
			2.09E-07	2.01E-07	3.89E-07	7.27E-07		
			2.12E-03	6.97E-04	1.70E-03	9.39E-04	8.67E-03	7.98E-03
1.71E-06	2.57E-07	1.53E-06	2.33E-06	3.14E-06	2.66E-07	2.29E-06	1.03E-06	1.66E-06
2.58E-03		2.01E-03	1.45E-02	8.66E-03	7.62E-04	4.99E-03	2.59E-03	8.47E-04
7.62E-05	3.57E-05		2.14E-05	2.51E-05	2.04E-05	2.51E-05	7.98E-05	5.19E-05
1.42E-04	7.22E-05							1.39E-04
					2.20E-02		1.01E-01	1.09E-01
								4.48E-03
								2.28E-02
				4.69E-03	2.48E-01		1.17E+00	4.74E-02

**36**	red	6.27E-08		9.55E-08				1.25E-07		1.91E-07				1.88E-07		2.86E-07			
**37**	black			3.53E-07	1.43E-03					7.06E-07	2.86E-03					1.06E-06	4.30E-03		
**38**	purple	6.80E-08	1.17E-04	6.33E-08		5.35E-03		1.36E-07	2.34E-04	1.27E-07		1.07E-02		2.04E-07	3.52E-04	1.90E-07		1.60E-02	
**39**	blue		1.23E-04	4.23E-07	6.69E-04	3.00E-03	4.02E-05		2.46E-04	8.46E-07	1.34E-03	6.01E-03	8.04E-05		3.69E-04	1.27E-06	2.01E-03	9.01E-03	1.21E-04
**40**	brown	7.32E-08	3.71E-04	9.64E-08		1.88E-05	2.98E-05	1.46E-07	7.41E-04	1.93E-07		3.76E-05	5.96E-05	2.20E-07	1.11E-03	2.89E-07		5.64E-05	8.94E-05
**41**	pink		1.33E-04	9.11E-07	1.77E-03	2.29E-05	2.51E-05		2.66E-04	1.82E-06	3.54E-03	4.58E-05	5.02E-05		3.99E-04	2.73E-06	5.30E-03	6.86E-05	7.53E-05

** Information provided by the manufacturer on the ink packaging – „May contain trace amounts of Nickel”.

Table S5 Systemic exposure dose (SED) [mg/kg body weight/day] calculated for the analyzed elements contained in tattoo inks used to create a 5 cm tattoo using the filling method.

No	Product purpose	Color	Brand	Comment	5 cm – Filling MIN											5 cm – Filling TYPICALLY											5 cm – Filling MAX										
					Pb	Cd	Zn	Cr	Ni	Cu	As	Co	Sb	Se	Mn	Pb	Cd	Zn	Cr	Ni	Cu	As	Co	Sb	Se	Mn	Pb	Cd	Zn	Cr	Ni	Cu	As	Co	Sb	Se	Mn
1	tattoo ink	white	1	**				2.03E-07	3.35E-04	4.39E-05	8.91E-05		1.53E-03						4.06E-07	6.70E-04	8.79E-05	1.78E-04	0.00E+00	3.07E-03						6.09E-07	1.01E-03	1.32E-04	2.67E-04		4.60E-03		
2		brown		**			6.67E-04	1.19E-06	1.25E-03	2.39E-05	7.10E-05	6.51E-02	2.28E-03	1.06E-02	1.27E-02			1.33E-03	2.38E-06	2.50E-03	4.79E-05	1.42E-04	1.30E-01	4.56E-03	2.13E-02	2.55E-02			2.00E-03	3.57E-06	3.76E-03	7.18E-05	2.13E-04	1.95E-01	6.83E-03	3.19E-02	3.82E-02
3		black				9.31E-08		2.49E-07	6.07E-04								1.86E-07		4.98E-07	1.21E-03								2.79E-07		7.48E-07	1.82E-03						
4		red						7.76E-08											1.55E-07											2.33E-07							
5		purple						1.53E-07	3.69E-04	5.86E-04	5.22E-05								3.05E-07	7.38E-04	1.17E-03	1.04E-04								4.58E-07	1.11E-03	1.76E-03	1.56E-04				
6		blue					2.70E-04	1.20E-07		2.75E-02								5.39E-04	2.41E-07		5.51E-02								8.09E-04	3.61E-07		8.26E-02					
7		orange		**				1.83E-07	4.31E-04	3.57E-05	3.99E-05								3.67E-07	8.61E-04	7.14E-05	7.99E-05								5.50E-07	1.29E-03	1.07E-04	1.20E-04				
8		pink		**				1.57E-07		3.94E-05	7.61E-05		1.31E-03						3.15E-07		7.88E-05	1.52E-04		2.62E-03						4.72E-07		1.18E-04	2.28E-04		3.93E-03		

17	16	15	14	13	12	11	10	9
green	pink	blue	purple	red	black	brown	yellow	green
			2					
						1.27E-06		
			3.28E-04		4.22E-04	1.22E-03		
1.01E-07	1.28E-07	1.20E-07	1.38E-06	1.95E-07	1.89E-06	1.74E-06	7.85E-08	1.42E-07
			5.21E-03		5.32E-03	1.63E-03		4.34E-04
5.89E-03	3.78E-05	3.69E-03	4.02E-05	2.42E-04	1.02E-05	2.80E-04		6.90E-03
	7.50E-05	6.57E-05				5.83E-05		4.52E-05
						4.28E-02		
	1.35E-03	1.34E-03				1.73E-03		
						7.68E-03		
					2.59E-03	5.81E-02		
						2.54E-06		
			6.57E-04		8.45E-04	2.44E-03		
2.03E-07	2.55E-07	2.40E-07	2.75E-06	3.91E-07	3.77E-06	3.48E-06	1.57E-07	2.84E-07
			1.04E-02		1.06E-02	3.26E-03		8.68E-04
1.18E-02	7.56E-05	7.38E-03	8.05E-05	4.84E-04	2.04E-05	5.60E-04		1.38E-02
	1.50E-04	1.31E-04				1.17E-04		9.03E-05
						8.56E-02		
	2.71E-03	2.69E-03				3.47E-03		
						1.54E-02		
					5.18E-03	1.16E-01		
						3.82E-06		
			9.85E-04		1.27E-03	3.66E-03		
3.04E-07	3.83E-07	3.60E-07	4.13E-06	5.86E-07	5.66E-06	5.23E-06	2.36E-07	4.26E-07
			1.56E-02		1.59E-02	4.89E-03		1.30E-03
1.77E-02	1.13E-04	1.11E-02	1.21E-04	7.26E-04	3.06E-05	8.41E-04		2.07E-02
	2.25E-04	1.97E-04				1.75E-04		1.35E-04
						1.28E-01		
	4.06E-03	4.03E-03				5.20E-03		
						2.30E-02		
					7.76E-03	1.74E-01		

26	25	24	23	22	21	20	19	18
green	pink	orange	blue	purple	black	white	orange	yellow
			**3**					
		9.30E-07						2.15E-06
9.91E-08			9.43E-08					
	1.99E-04	2.41E-04			5.04E-04	1.76E-04		4.38E-04
1.21E-07	9.56E-07	2.64E-06	2.05E-07	1.15E-07	1.47E-06	1.26E-07	9.50E-08	2.06E-06
	8.89E-03	5.83E-03			5.33E-03			4.88E-03
1.43E-03	3.99E-05	1.41E-05	5.93E-03	4.62E-05	1.09E-05	4.57E-05	1.36E-05	1.28E-05
5.72E-05	5.97E-05		5.36E-05	5.52E-05		1.72E-04		
		3.05E-03						
						1.94E-03		
		4.84E-03						3.47E-03
		1.86E-06						4.29E-06
1.98E-07			1.89E-07					
	3.98E-04	4.82E-04			1.01E-03	3.53E-04		8.76E-04
2.42E-07	1.91E-06	5.28E-06	4.09E-07	2.30E-07	2.93E-06	2.52E-07	1.90E-07	4.11E-06
	1.78E-02	1.17E-02			1.07E-02			9.77E-03
2.86E-03	7.97E-05	2.83E-05	1.19E-02	9.25E-05	2.18E-05	9.15E-05	2.72E-05	2.57E-05
1.14E-04	1.19E-04	0.00E+00	1.07E-04	1.10E-04		3.44E-04		
		6.10E-03						
						3.89E-03		
		9.68E-03						6.94E-03
		2.79E-06						6.44E-06
2.97E-07			2.83E-07					
	5.97E-04	7.22E-04			1.51E-03	5.29E-04		1.31E-03
3.64E-07	2.87E-06	7.92E-06	6.14E-07	3.46E-07	4.40E-06	3.78E-07	2.85E-07	6.17E-06
	2.67E-02	1.75E-02			1.60E-02			1.47E-02
4.29E-03	1.20E-04	4.24E-05	1.78E-02	1.39E-04	3.26E-05	1.37E-04	4.08E-05	3.85E-05
1.72E-04	1.79E-04		1.61E-04	1.65E-04		5.17E-04		
		9.15E-03						
						5.83E-03		
		1.45E-02						1.04E-02

35	34	33	32	31	30	29	28	27
**tattoo & permanent makeup ink**			**permanent makeup ink**					
yellow	white	orange	brown-black	black coffee	chocolate-brown	black	chocolate	yellow
	**7**		**6**		**5**	**4**		
						8.39E-06	2.34E-06	
			1.04E-07	1.00E-07	1.95E-07	3.64E-07		
		0.00E+00	1.06E-03	3.48E-04	8.51E-04	4.70E-04	4.33E-03	3.99E-03
8.57E-07	1.28E-07	7.65E-07	1.17E-06	1.57E-06	1.33E-07	1.14E-06	5.16E-07	8.31E-07
1.29E-03		1.01E-03	7.24E-03	4.33E-03	3.81E-04	2.50E-03	1.30E-03	4.23E-04
3.81E-05	1.79E-05		1.07E-05	1.25E-05	1.02E-05	1.26E-05	3.99E-05	2.59E-05
7.09E-05	3.61E-05							6.94E-05
					1.10E-02		5.05E-02	5.44E-02
								2.24E-03
								1.14E-02
				2.35E-03	1.24E-01		5.84E-01	2.37E-02
						1.68E-05	4.67E-06	
			2.09E-07	2.01E-07	3.89E-07	7.27E-07		
			2.12E-03	6.97E-04	1.70E-03	9.39E-04	8.67E-03	7.98E-03
1.71E-06	2.57E-07	1.53E-06	2.33E-06	3.14E-06	2.66E-07	2.29E-06	1.03E-06	1.66E-06
2.58E-03		2.01E-03	1.45E-02	8.66E-03	7.62E-04	4.99E-03	2.59E-03	8.47E-04
7.62E-05	3.57E-05		2.14E-05	2.51E-05	2.04E-05	2.51E-05	7.98E-05	5.19E-05
1.42E-04	7.22E-05							1.39E-04
					2.20E-02		1.01E-01	1.09E-01
								4.48E-03
								2.28E-02
				4.69E-03	2.48E-01		1.17E+00	4.74E-02
						2.52E-05	7.01E-06	
			3.13E-07	3.01E-07	5.84E-07	1.09E-06		
			3.18E-03	1.04E-03	2.55E-03	1.41E-03	1.30E-02	1.20E-02
2.57E-06	3.85E-07	2.29E-06	3.50E-06	4.70E-06	3.99E-07	3.43E-06	1.55E-06	2.49E-06
3.87E-03		3.02E-03	2.17E-02	1.30E-02	1.14E-03	7.49E-03	3.89E-03	1.27E-03
1.14E-04	5.36E-05		3.21E-05	3.76E-05	3.06E-05	3.77E-05	1.20E-04	7.78E-05
2.13E-04	1.08E-04							2.08E-04
					3.31E-02		1.52E-01	1.63E-01
								6.71E-03
								3.43E-02
				7.04E-03	3.71E-01		1.75E+00	7.10E-02

**36**	red	9.40E-08		1.43E-07				1.88E-07		2.86E-07				2.82E-07		4.30E-07			
**37**	black			5.30E-07	2.15E-03					1.06E-06	4.30E-03					1.59E-06	6.45E-03		
**38**	purple	1.02E-07	1.76E-04	9.49E-08		8.02E-03		2.04E-07	3.52E-04	1.90E-07		1.60E-02		3.06E-07	5.27E-04	2.85E-07		2.41E-02	
**39**	blue		1.84E-04	6.35E-07	1.00E-03	4.51E-03	6.03E-05		3.69E-04	1.27E-06	2.01E-03	9.01E-03	1.21E-04		5.53E-04	1.90E-06	3.01E-03	1.35E-02	1.81E-04
**40**	brown	1.10E-07	5.56E-04	1.45E-07		2.82E-05	4.47E-05	2.20E-07	1.11E-03	2.89E-07		5.64E-05	8.94E-05	3.29E-07	1.67E-03	4.34E-07		8.45E-05	1.34E-04
**41**	pink		2.00E-04	1.37E-06	2.65E-03	3.43E-05	3.76E-05		3.99E-04	2.73E-06	5.30E-03	6.86E-05	7.53E-05		5.99E-04	4.10E-06	7.95E-03	1.03E-04	1.13E-04

** Information provided by the manufacturer on the ink packaging – „May contain trace amounts of Nickel”.

Table S6 Margin of Safety (MoS) calculated for the analyzed elements contained in tattoo inks used to create a 5 cm tattoo using the contour method.

No	Product purpose	Color	Brand	Comment	5 cm – Contour MIN											5 cm – Contour TYPICALLY											5 cm – Contour MAX										
					Pb	Cd	Zn	Cr	Ni	Cu	As	Co	Sb	Se	Mn	Pb	Cd	Zn	Cr	Ni	Cu	As	Co	Sb	Se	Mn	Pb	Cd	Zn	Cr	Ni	Cu	As	Co	Sb	Se	Mn
1	tattoo ink	white	1	**				1.33E+03	2.15E+03	2.73E+01	1.82E+02		7.83E-01						6.65E+02	1.07E+03	1.37E+01	9.09E+01		3.91E-01						4.43E+02	7.16E+02	9.10E+00	6.06E+01		2.61E-01		
2		brown		**			8.09E+01	2.27E+02	5.75E+02	5.01E+01	2.28E+02	1.11E+02	5.27E-01	4.24E-02	3.30E-01			4.05E+01	1.13E+02	2.88E+02	2.51E+01	1.14E+02	5.53E+01	2.63E-01	2.12E-02	1.65E-01			2.70E+01	7.55E+01	1.92E+02	1.67E+01	7.60E+01	3.68E+01	1.76E-01	1.41E-02	1.10E-01
3		black				3.22E+02		1.08E+03	1.19E+03								1.61E+02		5.42E+02	5.93E+02								1.07E+02		3.61E+02	3.95E+02						
4		red						3.48E+03											1.74E+03											1.16E+03							
5		purple						1.77E+03	1.95E+03	2.05E+00	3.11E+02								8.85E+02	9.76E+02	1.02E+00	1.55E+02								5.90E+02	6.51E+02	6.82E-01	1.04E+02				
6		blue					2.00E+02	2.25E+03		4.36E-02								1.00E+02	1.12E+03		2.18E-02								6.67E+01	7.48E+02		1.45E-02					
7		orange		**				1.47E+03	1.67E+03	3.36E+01	4.06E+02								7.36E+02	8.36E+02	1.68E+01	2.03E+02								4.91E+02	5.57E+02	1.12E+01	1.35E+02				
8		pink		**				1.72E+03		3.04E+01	2.13E+02		9.16E-01						8.58E+02		1.52E+01	1.06E+02		4.58E-01						5.72E+02		1.01E+01	7.10E+01		3.05E-01		

**9**	green					1.90E+03	1.66E+03	1.74E-01	3.59E+02				
**10**	yellow					3.44E+03							
**11**	brown		2.83E+03		4.42E+01	1.55E+02	4.42E+02	4.28E+00	2.78E+02	1.68E+02	6.92E-01	5.86E-02	7.23E-02
**12**	black				1.28E+02	1.43E+02	1.35E+02	1.18E+02					1.62E+00
**13**	red					1.38E+03		4.96E+00					
**14**	purple	**2**			1.64E+02	1.96E+02	1.38E+02	2.98E+01					
**15**	blue					2.25E+03		3.25E-01	2.47E+02		8.92E-01		
**16**	pink					2.12E+03		3.18E+01	2.16E+02		8.86E-01		
**17**	green					2.66E+03		2.04E-01					

**9**	green					9.51E+02	8.30E+02	8.70E-02	1.79E+02				
**10**	yellow					1.72E+03							
**11**	brown		1.42E+03		2.21E+01	7.75E+01	2.21E+02	2.14E+00	1.39E+02	8.41E+01	3.46E-01	2.93E-02	3.61E-02
**12**	black				6.39E+01	7.16E+01	6.77E+01	5.89E+01					8.12E-01
**13**	red					6.91E+02		2.48E+00					
**14**	purple	**2**			8.22E+01	9.81E+01	6.91E+01	1.49E+01					
**15**	blue					1.12E+03		1.63E-01	1.23E+02		4.46E-01		
**16**	pink					1.06E+03		1.59E+01	1.08E+02		4.43E-01		
**17**	green					1.33E+03		1.02E-01					

**9**	green					6.34E+02	5.53E+02	5.80E-02	1.20E+02				
**10**	yellow					1.15E+03							
**11**	brown		9.44E+02		1.47E+01	5.17E+01	1.47E+02	1.43E+00	9.26E+01	5.61E+01	2.31E-01	1.95E-02	2.41E-02
**12**	black				4.26E+01	4.77E+01	4.52E+01	3.93E+01					5.41E-01
**13**	red					4.61E+02		1.65E+00					
**14**	purple	**2**			5.48E+01	6.54E+01	4.60E+01	9.94E+00					
**15**	blue					7.50E+02		1.08E-01	8.23E+01		2.97E-01		
**16**	pink					7.06E+02		1.06E+01	7.20E+01		2.95E-01		
**17**	green					8.88E+02		6.79E-02					

26	25	24	23	22	21	20	19	18
green	pink	orange	blue	purple	black	white	orange	yellow
			3					
		3.87E+03						1.68E+03
3.03E+02			3.18E+02					
	2.71E+02	2.24E+02			1.07E+02	3.06E+02		1.23E+02
2.23E+03	2.83E+02	1.02E+02	1.32E+03	2.34E+03	1.84E+02	2.14E+03	2.84E+03	1.31E+02
	8.10E+01	1.23E+02			1.35E+02			1.47E+02
8.39E-01	3.01E+01	8.49E+01	2.02E-01	2.60E+01	1.10E+02	2.62E+01	8.83E+01	9.34E+01
2.83E+02	2.71E+02		3.02E+02	2.94E+02		9.41E+01		
		2.36E+03						
						6.18E-01		
		8.68E-01						1.21E+00
		1.94E+03						8.39E+02
1.51E+02			1.59E+02					
	1.36E+02	1.12E+02			5.36E+01	1.53E+02		6.17E+01
1.11E+03	1.41E+02	5.11E+01	6.60E+02	1.17E+03	9.21E+01	1.07E+03	1.42E+03	6.57E+01
	4.05E+01	6.17E+01			6.76E+01			7.37E+01
4.19E-01	1.50E+01	4.24E+01	1.01E-01	1.30E+01	5.51E+01	1.31E+01	4.42E+01	4.67E+01
1.42E+02	1.36E+02		1.51E+02	1.47E+02		4.70E+01		
		1.18E+03						
						3.09E-01		
		4.34E-01						6.05E-01
		1.29E+03						5.59E+02
1.01E+02			1.06E+02					
	9.05E+01	7.48E+01			3.57E+01	1.02E+02		4.11E+01
7.43E+02	9.42E+01	3.41E+01	4.40E+02	7.81E+02	6.14E+01	7.14E+02	9.47E+02	4.38E+01
	2.70E+01	4.12E+01			4.51E+01			4.91E+01
2.80E-01	1.00E+01	2.83E+01	6.74E-02	8.65E+00	3.68E+01	8.74E+00	2.94E+01	3.11E+01
9.44E+01	9.04E+01		1.01E+02	9.79E+01		3.14E+01		
		7.87E+02						
						2.06E-01		
		2.89E-01						4.03E-01

35	34	33	32	31	30	29	28	27
tattoo & permanent makeup ink			permanent makeup ink					
yellow	white	orange	brown-black	black coffee	chocolate-brown	black	chocolate	yellow
	7		6		5	4		
						4.29E+02	1.54E+03	
			2.88E+02	2.99E+02	1.54E+02	8.25E+01		
			5.10E+01	1.55E+02	6.34E+01	1.15E+02	1.25E+01	1.35E+01
3.15E+02	2.10E+03	3.53E+02	2.32E+02	1.72E+02	2.03E+03	2.36E+02	5.24E+02	3.25E+02
5.58E+02		7.15E+02	9.95E+01	1.66E+02	1.89E+03	2.88E+02	5.56E+02	1.70E+03
3.15E+01	6.72E+01		1.12E+02	9.57E+01	1.18E+02	9.55E+01	3.01E+01	4.63E+01
2.28E+02	4.49E+02							2.34E+02
					6.53E+02		1.42E+02	1.32E+02
								5.36E-01
								3.94E-02
				1.79E+00	3.39E-02		7.19E-03	1.77E-01
						2.15E+02	7.70E+02	
			1.44E+02	1.50E+02	7.71E+01	4.13E+01		
			2.55E+01	7.75E+01	3.17E+01	5.75E+01	6.23E+00	6.77E+00
1.58E+02	1.05E+03	1.77E+02	1.16E+02	8.61E+01	1.01E+03	1.18E+02	2.62E+02	1.62E+02
2.79E+02		3.57E+02	4.97E+01	8.32E+01	9.45E+02	1.44E+02	2.78E+02	8.51E+02
1.57E+01	3.36E+01		5.61E+01	4.78E+01	5.89E+01	4.77E+01	1.50E+01	2.31E+01
1.14E+02	2.25E+02							1.17E+02
					3.27E+02		7.12E+01	6.61E+01
								2.68E-01
								1.97E-02
				8.95E-01	1.70E-02		3.60E-03	8.87E-02
						1.43E+02	5.14E+02	
			9.59E+01	9.97E+01	5.14E+01	2.75E+01		
			1.70E+01	5.17E+01	2.11E+01	3.83E+01	4.15E+00	4.51E+00
1.05E+02	7.01E+02	1.18E+02	7.72E+01	5.74E+01	6.76E+02	7.88E+01	1.75E+02	1.08E+02
1.86E+02		2.38E+02	3.32E+01	5.54E+01	6.30E+02	9.61E+01	1.85E+02	5.67E+02
1.05E+01	2.24E+01		3.74E+01	3.19E+01	3.92E+01	3.18E+01	1.00E+01	1.54E+01
7.61E+01	1.50E+02							7.78E+01
					2.18E+02		4.75E+01	4.41E+01
								1.79E-01
								1.31E-02
				5.97E-01	1.13E-02		2.40E-03	5.91E-02

**36**	red	3.19E+02		1.89E+03				1.60E+02		9.43E+02				1.06E+02		6.28E+02			
**37**	black			5.10E+02	3.35E+02					2.55E+02	1.68E+02					1.70E+02	1.12E+02		
**38**	purple	2.94E+02	3.07E+02	2.84E+03		1.50E-01		1.47E+02	1.54E+02	1.42E+03		7.48E-02		9.81E+01	1.02E+02	9.48E+02		4.99E-02	
**39**	blue		2.93E+02	4.26E+02	7.18E+02	2.66E-01	2.69E+02		1.46E+02	2.13E+02	3.59E+02	1.33E-01	1.34E+02		9.76E+01	1.42E+02	2.39E+02	8.88E-02	8.95E+01
**40**	brown	2.73E+02	9.71E+01	1.87E+03		4.26E+01	3.63E+02	1.37E+02	4.86E+01	9.33E+02		2.13E+01	1.81E+02	9.11E+01	3.24E+01	6.22E+02		1.42E+01	1.21E+02
**41**	pink		2.71E+02	1.98E+02	2.72E+02	3.50E+01	4.30E+02		1.35E+02	9.88E+01	1.36E+02	1.75E+01	2.15E+02		9.02E+01	6.58E+01	9.05E+01	1.17E+01	1.43E+02

** Information provided by the manufacturer on the ink packaging – „May contain trace amounts of Nickel".

Table S7 Margin of Safety (MoS) calculated for the analyzed elements contained in tattoo inks used to create a 5 cm tattoo using the realism method.

No	Product purpose	Color	Brand	Comment	5 cm – Realism MIN										
					Pb	Cd	Zn	Cr	Ni	Cu	As	Co	Sb	Se	Mn
1	tattoo ink	white	1	**				6.65E+02	1.07E+03	1.37E+01	9.09E+01		3.91E-01		
2		brown		**			4.05E+01	1.13E+02	2.88E+02	2.51E+01	1.14E+02	5.53E+01	2.63E-01	2.12E-02	1.65E-01
3		black				1.61E+02		5.42E+02	5.93E+02						
4		red						1.74E+03							
5		purple						8.85E+02	9.76E+02	1.02E+00	1.55E+02				
6		blue					1.00E+02	1.12E+03		2.18E-02					
7		orange		**				7.36E+02	8.36E+02	1.68E+01	2.03E+02				
8		pink		**				8.58E+02		1.52E+01	1.06E+02		4.58E-01		

No	Product purpose	Color	Brand	Comment	5 cm – Realism TYPICALLY										
					Pb	Cd	Zn	Cr	Ni	Cu	As	Co	Sb	Se	Mn
1	tattoo ink	white	1	**				3.32E+02	5.37E+02	6.83E+00	4.55E+01		1.96E-01		
2		brown		**			2.02E+01	5.67E+01	1.44E+02	1.25E+01	5.70E+01	2.76E+01	1.32E-01	1.06E-02	8.25E-02
3		black				8.06E+01		2.71E+02	2.96E+02						
4		red						8.70E+02							
5		purple						4.43E+02	4.88E+02	5.12E-01	7.77E+01				
6		blue					5.01E+01	5.61E+02		1.09E-02					
7		orange		**				3.68E+02	4.18E+02	8.41E+00	1.01E+02				
8		pink		**				4.29E+02		7.61E+00	5.32E+01		2.29E-01		

No	Product purpose	Color	Brand	Comment	5 cm – Realism MAX										
					Pb	Cd	Zn	Cr	Ni	Cu	As	Co	Sb	Se	Mn
1	tattoo ink	white	1	**				2.22E+02	3.58E+02	4.55E+00	3.03E+01		1.30E-01		
2		brown		**			1.35E+01	3.78E+01	9.58E+01	8.36E+00	3.80E+01	1.84E+01	8.78E-02	7.06E-03	5.50E-02
3		black				5.37E+01		1.81E+02	1.98E+02						
4		red						5.80E+02							
5		purple						2.95E+02	3.25E+02	3.41E-01	5.18E+01				
6		blue					3.34E+01	3.74E+02		7.26E-03					
7		orange		**				2.45E+02	2.79E+02	5.61E+00	6.76E+01				
8		pink		**				2.86E+02		5.07E+00	3.55E+01		1.53E-01		

**9**	green				9.51E+02	8.30E+02	8.70E-02	1.79E+02							4.75E+02	4.15E+02	4.35E-02	8.97E+01							3.17E+02	2.77E+02	2.90E-02	5.98E+01				
**10**	yellow				1.72E+03										8.60E+02										5.73E+02							
**11**	brown		1.42E+03	2.21E+01	7.75E+01	2.21E+02	2.14E+00	1.39E+02	8.41E+01	3.46E-01	2.93E-02	3.61E-02	7.08E+02	1.11E+01	3.87E+01	1.10E+02	1.07E+00	6.94E+01	4.21E+01	1.73E-01	1.47E-02	1.81E-02	4.72E+02	7.37E+00	2.58E+01	7.36E+01	7.14E-01	4.63E+01	2.80E+01	1.15E-01	9.77E-03	1.20E-02
**12**	black			6.39E+01	7.16E+01	6.77E+01	5.89E+01					8.12E-01		3.20E+01	3.58E+01	3.39E+01	2.95E+01					4.06E-01		2.13E+01	2.39E+01	2.26E+01	1.96E+01					2.71E-01
**13**	red				6.91E+02		2.48E+00								3.45E+02		1.24E+00								2.30E+02		8.27E-01					
**14**	purple	**2**		8.22E+01	9.81E+01	6.91E+01	1.49E+01							4.11E+01	4.90E+01	3.45E+01	7.46E+00							2.74E+01	3.27E+01	2.30E+01	4.97E+00					
**15**	blue				1.12E+03		1.63E-01	1.23E+02		4.46E-01					5.62E+02		8.13E-02	6.17E+01		2.23E-01					3.75E+02		5.42E-02	4.11E+01		1.49E-01		
**16**	pink				1.06E+03		1.59E+01	1.08E+02		4.43E-01					5.29E+02		7.94E+00	5.40E+01		2.21E-01					3.53E+02		5.29E+00	3.60E+01		1.48E-01		
**17**	green				1.33E+03		1.02E-01								6.66E+02		5.09E-02								4.44E+02		3.39E-02					

**18**	yellow		8.39E+02		6.17E+01	6.57E+01	7.37E+01	4.67E+01				6.05E-01	4.19E+02		3.08E+01	3.28E+01	3.68E+01	2.34E+01				3.02E-01	2.80E+02		2.06E+01	2.19E+01	2.46E+01	1.56E+01				2.02E-01
**19**	orange					1.42E+03		4.42E+01								7.10E+02		2.21E+01								4.73E+02		1.47E+01				
**20**	white				1.53E+02	1.07E+03		1.31E+01	4.70E+01		3.09E-01				7.66E+01	5.35E+02		6.56E+00	2.35E+01		1.54E-01				5.10E+01	3.57E+02		4.37E+00	1.57E+01		1.03E-01	
**21**	black				5.36E+01	9.21E+01	6.76E+01	5.51E+01							2.68E+01	4.61E+01	3.38E+01	2.76E+01							1.79E+01	3.07E+01	2.25E+01	1.84E+01				
**22**	purple					1.17E+03		1.30E+01	1.47E+02							5.86E+02		6.49E+00	7.34E+01							3.91E+02		4.33E+00	4.90E+01			
**23**	blue	**3**		1.59E+02		6.60E+02		1.01E-01	1.51E+02					7.96E+01		3.30E+02		5.05E-02	7.56E+01					5.30E+01		2.20E+02		3.37E-02	5.04E+01			
**24**	orange		1.94E+03		1.12E+02	5.11E+01	6.17E+01	4.24E+01		1.18E+03		4.34E-01	9.68E+02		5.61E+01	2.56E+01	3.09E+01	2.12E+01		5.90E+02		2.17E-01	6.45E+02		3.74E+01	1.70E+01	2.06E+01	1.41E+01		3.93E+02		1.45E-01
**25**	pink				1.36E+02	1.41E+02	4.05E+01	1.50E+01	1.36E+02						6.78E+01	7.06E+01	2.03E+01	7.52E+00	6.78E+01						4.52E+01	4.71E+01	1.35E+01	5.02E+00	4.52E+01			
**26**	green			1.51E+02		1.11E+03		4.19E-01	1.42E+02					7.57E+01		5.57E+02		2.10E-01	7.08E+01					5.05E+01		3.71E+02		1.40E-01	4.72E+01			

35	34	33	32	31	30	29	28	27
**tattoo & permanent makeup ink**			**permanent makeup ink**					
yellow	white	orange	brown-black	black coffee	chocolate-brown	black	chocolate	yellow
	**7**		**6**		**5**	**4**		
						2.15E+02	7.70E+02	
			1.44E+02	1.50E+02	7.71E+01	4.13E+01		
			2.55E+01	7.75E+01	3.17E+01	5.75E+01	6.23E+00	6.77E+00
1.58E+02	1.05E+03	1.77E+02	1.16E+02	8.61E+01	1.01E+03	1.18E+02	2.62E+02	1.62E+02
2.79E+02		3.57E+02	4.97E+01	8.32E+01	9.45E+02	1.44E+02	2.78E+02	8.51E+02
1.57E+01	3.36E+01		5.61E+01	4.78E+01	5.89E+01	4.77E+01	1.50E+01	2.31E+01
1.14E+02	2.25E+02							1.17E+02
					3.27E+02		7.12E+01	6.61E+01
								2.68E-01
								1.97E-02
				8.95E-01	1.70E-02		3.60E-03	8.87E-02
						1.07E+02	3.85E+02	
			7.19E+01	7.48E+01	3.85E+01	2.06E+01		
			1.27E+01	3.88E+01	1.59E+01	2.87E+01	3.12E+00	3.38E+00
7.88E+01	5.25E+02	8.83E+01	5.79E+01	4.31E+01	5.07E+02	5.91E+01	1.31E+02	8.12E+01
1.40E+02		1.79E+02	2.49E+01	4.16E+01	4.72E+02	7.21E+01	1.39E+02	4.25E+02
7.87E+00	1.68E+01		2.80E+01	2.39E+01	2.94E+01	2.39E+01	7.52E+00	1.16E+01
5.71E+01	1.12E+02							5.84E+01
					1.63E+02		3.56E+01	3.31E+01
								1.34E-01
								9.85E-03
				4.47E-01	8.48E-03		1.80E-03	4.43E-02
						7.16E+01	2.57E+02	
			4.80E+01	4.98E+01	2.57E+01	1.38E+01		
			8.49E+00	2.58E+01	1.06E+01	1.92E+01	2.08E+00	2.26E+00
5.25E+01	3.50E+02	5.88E+01	3.86E+01	2.87E+01	3.38E+02	3.94E+01	8.73E+01	5.41E+01
9.30E+01		1.19E+02	1.66E+01	2.77E+01	3.15E+02	4.81E+01	9.26E+01	2.84E+02
5.25E+00	1.12E+01		1.87E+01	1.59E+01	1.96E+01	1.59E+01	5.01E+00	7.71E+00
3.81E+01	7.48E+01							3.89E+01
					1.09E+02		2.37E+01	2.20E+01
								8.94E-02
								6.57E-03
				2.98E-01	5.65E-03		1.20E-03	2.96E-02

**36**	red	1.60E+02		9.43E+02				7.98E+01		4.71E+02				5.32E+01		3.14E+02			
**37**	black			2.55E+02	1.68E+02					1.27E+02	8.38E+01					8.49E+01	5.59E+01		
**38**	purple	1.47E+02	1.54E+02	1.42E+03		7.48E-02		7.36E+01	7.68E+01	7.11E+02		3.74E-02		4.90E+01	5.12E+01	4.74E+02		2.49E-02	
**39**	blue		1.46E+02	2.13E+02	3.59E+02	1.33E-01	1.34E+02		7.32E+01	1.06E+02	1.79E+02	6.66E-02	6.72E+01		4.88E+01	7.09E+01	1.20E+02	4.44E-02	4.48E+01
**40**	brown	1.37E+02	4.86E+01	9.33E+02		2.13E+01	1.81E+02	6.83E+01	2.43E+01	4.67E+02		1.06E+01	9.06E+01	4.55E+01	1.62E+01	3.11E+02		7.10E+00	6.04E+01
**41**	pink		1.35E+02	9.88E+01	1.36E+02	1.75E+01	2.15E+02		6.76E+01	4.94E+01	6.79E+01	8.74E+00	1.08E+02		4.51E+01	3.29E+01	4.53E+01	5.83E+00	7.17E+01

** Information provided by the manufacturer on the ink packaging – „May contain trace amounts of Nickel”.

Table S8 Margin of Safety (MoS) calculated for the analyzed elements contained in tattoo inks used to create a 5 cm tattoo using the filling method.

No	Product purpose	Color	Brand	Comment	5 cm – Filling MIN											5 cm – Filling TYPICALLY											5 cm – Filling MAX										
					Pb	Cd	Zn	Cr	Ni	Cu	As	Co	Sb	Se	Mn	Pb	Cd	Zn	Cr	Ni	Cu	As	Co	Sb	Se	Mn	Pb	Cd	Zn	Cr	Ni	Cu	As	Co	Sb	Se	Mn
1	tattoo ink	white	1	**				4.43E+02	7.16E+02	9.10E+00	6.06E+01		2.61E-01						2.22E+02	3.58E+02	4.55E+00	3.03E+01		1.30E-01						1.48E+02	2.39E+02	3.03E+00	2.02E+01		8.70E-02		
2		brown		**			2.70E+01	7.55E+01	1.92E+02	1.67E+01	7.60E+01	3.68E+01	1.76E-01	1.41E-02	1.10E-01			1.35E+01	3.78E+01	9.58E+01	8.36E+00	3.80E+01	1.84E+01	8.78E-02	7.06E-03	5.50E-02			8.99E+00	2.52E+01	6.39E+01	5.57E+00	2.53E+01	1.23E+01	5.85E-02	4.71E-03	3.67E-02
3		black				1.07E+02		3.61E+02	3.95E+02								5.37E+01		1.81E+02	1.98E+02								3.58E+01		1.20E+02	1.32E+02						
4		red						1.16E+03											5.80E+02											3.86E+02							
5		purple						5.90E+02	6.51E+02	6.82E-01	1.04E+02								2.95E+02	3.25E+02	3.41E-01	5.18E+01								1.97E+02	2.17E+02	2.27E-01	3.45E+01				
6		blue					6.67E+01	7.48E+02		1.45E-02								3.34E+01	3.74E+02		7.26E-03								2.22E+01	2.49E+02		4.84E-03					
7		orange		**				4.91E+02	5.57E+02	1.12E+01	1.35E+02								2.45E+02	2.79E+02	5.61E+00	6.76E+01								1.64E+02	1.86E+02	3.74E+00	4.51E+01				
8		pink		**				5.72E+02		1.01E+01	7.10E+01		3.05E-01						2.86E+02		5.07E+00	3.55E+01		1.53E-01						1.91E+02		3.38E+00	2.37E+01		1.02E-01		

9	green				6.34E+02	5.53E+02	5.80E-02	1.20E+02				
10	yellow				1.15E+03							
11	brown		9.44E+02	1.47E+01	5.17E+01	1.47E+02	1.43E+00	9.26E+01	5.61E+01	2.31E-01	1.95E-02	2.41E-02
12	black			4.26E+01	4.77E+01	4.52E+01	3.93E+01					5.41E-01
13	red				4.61E+02		1.65E+00					
14	purple	2		5.48E+01	6.54E+01	4.60E+01	9.94E+00					
15	blue				7.50E+02		1.08E-01	8.23E+01		2.97E-01		
16	pink				7.06E+02		1.06E+01	7.20E+01		2.95E-01		
17	green				8.88E+02		6.79E-02					

9	green			3.17E+02	2.77E+02	2.90E-02	5.98E+01				
10	yellow			5.73E+02							
11	brown	4.72E+02	7.37E+00	2.58E+01	7.36E+01	7.14E-01	4.63E+01	2.80E+01	1.15E-01	9.77E-03	1.20E-02
12	black		2.13E+01	2.39E+01	2.26E+01	1.96E+01					2.71E-01
13	red			2.30E+02		8.27E-01					
14	purple		2.74E+01	3.27E+01	2.30E+01	4.97E+00					
15	blue			3.75E+02		5.42E-02	4.11E+01		1.49E-01		
16	pink			3.53E+02		5.29E+00	3.60E+01		1.48E-01		
17	green			4.44E+02		3.39E-02					

9	green			2.11E+02	1.84E+02	1.93E-02	3.99E+01				
10	yellow			3.82E+02							
11	brown	3.15E+02	4.91E+00	1.72E+01	4.91E+01	4.76E-01	3.09E+01	1.87E+01	7.69E-02	6.51E-03	8.03E-03
12	black		1.42E+01	1.59E+01	1.51E+01	1.31E+01					1.80E-01
13	red			1.54E+02		5.51E-01					
14	purple		1.83E+01	2.18E+01	1.53E+01	3.31E+00					
15	blue			2.50E+02		3.61E-02	2.74E+01		9.92E-02		
16	pink			2.35E+02		3.53E+00	2.40E+01		9.84E-02		
17	green			2.96E+02		2.26E-02					

26	25	24	23	22	21	20	19	18
green	pink	orange	blue	purple	black	white	orange	yellow
			3					
		1.29E+03						5.59E+02
1.01E+02			1.06E+02					
	9.05E+01	7.48E+01			3.57E+01	1.02E+02		4.11E+01
7.43E+02	9.42E+01	3.41E+01	4.40E+02	7.81E+02	6.14E+01	7.14E+02	9.47E+02	4.38E+01
	2.70E+01	4.12E+01			4.51E+01			4.91E+01
2.80E-01	1.00E+01	2.83E+01	6.74E-02	8.65E+00	3.68E+01	8.74E+00	2.94E+01	3.11E+01
9.44E+01	9.04E+01		1.01E+02	9.79E+01		3.14E+01		
		7.87E+02						
						2.06E-01		
		2.89E-01						4.03E-01
		6.45E+02						2.80E+02
5.05E+01			5.30E+01					
	4.52E+01	3.74E+01			1.79E+01	5.10E+01		2.06E+01
3.71E+02	4.71E+01	1.70E+01	2.20E+02	3.91E+02	3.07E+01	3.57E+02	4.73E+02	2.19E+01
	1.35E+01	2.06E+01			2.25E+01			2.46E+01
1.40E-01	5.02E+00	1.41E+01	3.37E-02	4.33E+00	1.84E+01	4.37E+00	1.47E+01	1.56E+01
4.72E+01	4.52E+01		5.04E+01	4.90E+01		1.57E+01		
		3.93E+02						
						1.03E-01		
		1.45E-01						2.02E-01
		4.30E+02						1.86E+02
3.36E+01			3.54E+01					
	3.02E+01	2.49E+01			1.19E+01	3.40E+01		1.37E+01
2.48E+02	3.14E+01	1.14E+01	1.47E+02	2.60E+02	2.05E+01	2.38E+02	3.16E+02	1.46E+01
	9.00E+00	1.37E+01			1.50E+01			1.64E+01
9.32E-02	3.34E+00	9.43E+00	2.25E-02	2.88E+00	1.23E+01	2.91E+00	9.81E+00	1.04E+01
3.15E+01	3.01E+01		3.36E+01	3.26E+01		1.05E+01		
		2.62E+02						
						6.86E-02		
		9.65E-02						1.34E-01

35	34	33	32	31	30	29	28	27
tattoo & permanent makeup ink			permanent makeup ink					
yellow	white	orange	brown-black	black coffee	chocolate-brown	black	chocolate	yellow
	7		6		5	4		
						1.43E+02	5.14E+02	
			9.59E+01	9.97E+01	5.14E+01	2.75E+01		
			1.70E+01	5.17E+01	2.11E+01	3.83E+01	4.15E+00	4.51E+00
1.05E+02	7.01E+02	1.18E+02	7.72E+01	5.74E+01	6.76E+02	7.88E+01	1.75E+02	1.08E+02
1.86E+02		2.38E+02	3.32E+01	5.54E+01	6.30E+02	9.61E+01	1.85E+02	5.67E+02
1.05E+01	2.24E+01		3.74E+01	3.19E+01	3.92E+01	3.18E+01	1.00E+01	1.54E+01
7.61E+01	1.50E+02							7.78E+01
					2.18E+02		4.75E+01	4.41E+01
								1.79E-01
								1.31E-02
				5.97E-01	1.13E-02		2.40E-03	5.91E-02
						7.16E+01	2.57E+02	
			4.80E+01	4.98E+01	2.57E+01	1.38E+01		
			8.49E+00	2.58E+01	1.06E+01	1.92E+01	2.08E+00	2.26E+00
5.25E+01	3.50E+02	5.88E+01	3.86E+01	2.87E+01	3.38E+02	3.94E+01	8.73E+01	5.41E+01
9.30E+01		1.19E+02	1.66E+01	2.77E+01	3.15E+02	4.81E+01	9.26E+01	2.84E+02
5.25E+00	1.12E+01		1.87E+01	1.59E+01	1.96E+01	1.59E+01	5.01E+00	7.71E+00
3.81E+01	7.48E+01							3.89E+01
					1.09E+02		2.37E+01	2.20E+01
								8.94E-02
								6.57E-03
				2.98E-01	5.65E-03		1.20E-03	2.96E-02
						4.77E+01	1.71E+02	
			3.20E+01	3.32E+01	1.71E+01	9.17E+00		
			5.66E+00	1.72E+01	7.05E+00	1.28E+01	1.38E+00	1.50E+00
3.50E+01	2.34E+02	3.92E+01	2.57E+01	1.91E+01	2.25E+02	2.63E+01	5.82E+01	3.61E+01
6.20E+01		7.94E+01	1.11E+01	1.85E+01	2.10E+02	3.20E+01	6.17E+01	1.89E+02
3.50E+00	7.46E+00		1.25E+01	1.06E+01	1.31E+01	1.06E+01	3.34E+00	5.14E+00
2.54E+01	4.99E+01							2.59E+01
					7.26E+01		1.58E+01	1.47E+01
								5.96E-02
								4.38E-03
				1.99E-01	3.77E-03		7.99E-04	1.97E-02

**36**	red	1.06E+02		6.28E+02				5.32E+01		3.14E+02				3.55E+01		2.09E+02			
**37**	black			1.70E+02	1.12E+02					8.49E+01	5.59E+01					5.66E+01	3.72E+01		
**38**	purple	9.81E+01	1.02E+02	9.48E+02		4.99E-02		4.90E+01	5.12E+01	4.74E+02		2.49E-02		3.27E+01	3.41E+01	3.16E+02		1.66E-02	
**39**	blue		9.76E+01	1.42E+02	2.39E+02	8.88E-02	8.95E+01		4.88E+01	7.09E+01	1.20E+02	4.44E-02	4.48E+01		3.25E+01	4.73E+01	7.97E+01	2.96E-02	2.98E+01
**40**	brown	9.11E+01	3.24E+01	6.22E+02		1.42E+01	1.21E+02	4.55E+01	1.62E+01	3.11E+02		7.10E+00	6.04E+01	3.04E+01	1.08E+01	2.07E+02		4.73E+00	4.03E+01
**41**	pink		9.02E+01	6.58E+01	9.05E+01	1.17E+01	1.43E+02		4.51E+01	3.29E+01	4.53E+01	5.83E+00	7.17E+01		3.01E+01	2.19E+01	3.02E+01	3.89E+00	4.78E+01

** Information provided by the manufacturer on the ink packaging – „May contain trace amounts of Nickel”.

Table S9 Hazard Quotient (HQ) calculated for the analyzed elements contained in tattoo inks used to create a 5 cm tattoo using the contour method.

No	Product purpose	Color	Brand	Comment	5 cm – Contour MIN											5 cm – Contour TYPICALLY											5 cm – Contour MAX										
					Pb	Cd	Zn	Cr	Ni	Cu	As	Co	Sb	Se	Mn	Pb	Cd	Zn	Cr	Ni	Cu	As	Co	Sb	Se	Mn	Pb	Cd	Zn	Cr	Ni	Cu	As	Co	Sb	Se	Mn
1	tattoo ink	white	1	**				7.52E-02	1.40E-01	3.66E-01	1.65E-02		1.28E+03						1.50E-01	2.79E-01	7.32E-01	3.30E-02		2.56E+03						2.26E-01	4.19E-01	1.10E+00	4.95E-02		3.83E+03		
2		brown		**			3.71E-02	4.41E-01	5.22E-01	1.99E-01	1.32E-02	9.05E-01	1.90E+03	7.08E+01	3.03E+00			7.41E-02	8.82E-01	1.04E+00	3.99E-01	2.63E-02	1.81E+00	3.80E+03	1.42E+02	6.06E+00			1.11E-01	1.32E+00	1.57E+00	5.98E-01	3.95E-02	2.71E+00	5.70E+03	2.13E+02	9.09E+00
3		black				3.10E-02		9.23E-02	2.53E-01								6.20E-02		1.85E-01	5.06E-01								9.31E-02		2.77E-01	7.59E-01						
4		red						2.88E-02											5.75E-02											8.63E-02							
5		purple						5.65E-02	1.54E-01	4.89E+00	9.66E-03								1.13E-01	3.07E-01	9.77E+00	1.93E-02								1.69E-01	4.61E-01	1.47E+01	2.90E-02				
6		blue					1.50E-02	4.45E-02		2.29E+02								3.00E-02	8.91E-02		4.59E+02								4.49E-02	1.34E-01		6.88E+02					
7		orange		**				6.79E-02	1.79E-01	2.97E-01	7.40E-03								1.36E-01	3.59E-01	5.95E-01	1.48E-02								2.04E-01	5.38E-01	8.92E-01	2.22E-02				
8		pink		**				5.83E-02		3.28E-01	1.41E-02		1.09E+03						1.17E-01		6.57E-01	2.82E-02		2.18E+03						1.75E-01		9.85E-01	4.23E-02		3.27E+03		

9	green					5.26E-02	1.81E-01	5.75E+01	8.36E-03				
10	yellow					2.91E-02							
11	brown		3.53E-02		6.79E-02	6.45E-01	6.79E-01	2.34E+00	1.08E-02	5.94E-01	1.45E+03	5.12E+01	1.38E+01
12	black				2.35E-02	6.98E-01	2.21E+00	8.49E-02					6.16E-01
13	red					7.24E-02		2.02E+00					
14	purple	2			1.82E-02	5.10E-01	2.17E+00	3.35E-01					
15	blue					4.45E-02		3.07E+01	1.22E-02		1.12E+03		
16	pink					4.72E-02		3.15E-01	1.39E-02		1.13E+03		
17	green					3.75E-02		4.91E+01					

9	green				1.05E-01	3.61E-01	1.15E+02	1.67E-02				
10	yellow				5.82E-02							
11	brown	7.07E-02		1.36E-01	1.29E+00	1.36E+00	4.67E+00	2.16E-02	1.19E+00	2.89E+03	1.02E+02	2.77E+01
12	black			4.69E-02	1.40E+00	4.43E+00	1.70E-01					1.23E+00
13	red				1.45E-01		4.03E+00					
14	purple			3.65E-02	1.02E+00	4.34E+00	6.71E-01					
15	blue				8.89E-02		6.15E+01	2.43E-02		2.24E+03		
16	pink				9.45E-02		6.30E-01	2.78E-02		2.26E+03		
17	green				7.51E-02		9.82E+01					

9	green				1.58E-01	5.42E-01	1.72E+02	2.51E-02				
10	yellow				8.72E-02							
11	brown	1.06E-01		2.04E-01	1.94E+00	2.04E+00	7.01E+00	3.24E-02	1.78E+00	4.34E+03	1.54E+02	4.15E+01
12	black			7.04E-02	2.09E+00	6.64E+00	2.55E-01					1.85E+00
13	red				2.17E-01		6.05E+00					
14	purple			5.47E-02	1.53E+00	6.52E+00	1.01E+00					
15	blue				1.33E-01		9.22E+01	3.65E-02		3.36E+03		
16	pink				1.42E-01		9.45E-01	4.17E-02		3.39E+03		
17	green				1.13E-01		1.47E+02					

26	25	24	23	22	21	20	19	18
green	pink	orange	blue	purple	black	white	orange	yellow
			3					
		2.58E-02						5.96E-02
3.30E-02			3.14E-02					
	1.11E-02	1.34E-02			2.80E-02	9.80E-03		2.43E-02
4.49E-02	3.54E-01	9.78E-01	7.58E-02	4.27E-02	5.43E-01	4.67E-02	3.52E-02	7.61E-01
	3.70E+00	2.43E+00			2.22E+00			2.04E+00
1.19E+01	3.32E-01	1.18E-01	4.95E+01	3.85E-01	9.07E-02	3.81E-01	1.13E-01	1.07E-01
1.06E-02	1.11E-02		9.92E-03	1.02E-02		3.19E-02		
		4.24E-02						
						1.62E+03		
		1.15E+00						8.26E-01
		5.17E-02						1.19E-01
6.61E-02			6.29E-02					
	2.21E-02	2.68E-02			5.60E-02	1.96E-02		4.86E-02
8.98E-02	7.08E-01	1.96E+00	1.52E-01	8.53E-02	1.09E+00	9.34E-02	7.04E-02	1.52E+00
	7.40E+00	4.86E+00			4.44E+00			4.07E+00
2.38E+01	6.64E-01	2.36E-01	9.89E+01	7.71E-01	1.81E-01	7.62E-01	2.26E-01	2.14E-01
2.12E-02	2.21E-02		1.98E-02	2.04E-02		6.38E-02		
		8.47E-02						
						3.24E+03		
		2.30E+00						1.65E+00
		7.75E-02						1.79E-01
9.91E-02			9.43E-02					
	3.32E-02	4.01E-02			8.39E-02	2.94E-02		7.30E-02
1.35E-01	1.06E+00	2.93E+00	2.27E-01	1.28E-01	1.63E+00	1.40E-01	1.06E-01	2.28E+00
	1.11E+01	7.29E+00			6.66E+00			6.11E+00
3.58E+01	9.97E-01	3.54E-01	1.48E+02	1.16E+00	2.72E-01	1.14E+00	3.40E-01	3.21E-01
3.18E-02	3.32E-02		2.98E-02	3.06E-02		9.57E-02		
		1.27E-01						
						4.86E+03		
		3.46E+00						2.48E+00

35	34	33	32	31	30	29	28	27
**tattoo & permanent makeup ink**			**permanent makeup ink**					
yellow	white	orange	brown-black	black coffee	chocolate-brown	black	chocolate	yellow
	**7**		**6**		**5**	**4**		
						2.33E-01	6.49E-02	
			3.48E-02	3.34E-02	6.49E-02	1.21E-01		
			5.89E-02	1.93E-02	4.73E-02	2.61E-02	2.41E-01	2.22E-01
3.17E-01	4.76E-02	2.83E-01	4.32E-01	5.81E-01	4.93E-02	4.23E-01	1.91E-01	3.08E-01
5.38E-01		4.20E-01	3.02E+00	1.80E+00	1.59E-01	1.04E+00	5.40E-01	1.76E-01
3.18E-01	1.49E-01		8.91E-02	1.05E-01	8.50E-02	1.05E-01	3.33E-01	2.16E-01
1.31E-02	6.68E-03							1.28E-02
					1.53E-01		7.02E-01	7.56E-01
								1.87E+03
								7.61E+01
				5.59E-01	2.95E+01		1.39E+02	5.64E+00
						4.66E-01	1.30E-01	
			6.95E-02	6.69E-02	1.30E-01	2.42E-01		
			1.18E-01	3.87E-02	9.46E-02	5.22E-02	4.81E-01	4.43E-01
6.35E-01	9.52E-02	5.67E-01	8.64E-01	1.16E+00	9.86E-02	8.46E-01	3.82E-01	6.16E-01
1.08E+00		8.39E-01	6.03E+00	3.61E+00	3.18E-01	2.08E+00	1.08E+00	3.53E-01
6.35E-01	2.98E-01		1.78E-01	2.09E-01	1.70E-01	2.09E-01	6.65E-01	4.32E-01
2.63E-02	1.34E-02							2.57E-02
					3.06E-01		1.40E+00	1.51E+00
								3.73E+03
								1.52E+02
				1.12E+00	5.90E+01		2.78E+02	1.13E+01
						6.99E-01	1.95E-01	
			1.04E-01	1.00E-01	1.95E-01	3.64E-01		
			1.77E-01	5.80E-02	1.42E-01	7.83E-02	7.22E-01	6.65E-01
9.52E-01	1.43E-01	8.50E-01	1.30E+00	1.74E+00	1.48E-01	1.27E+00	5.73E-01	9.24E-01
1.61E+00		1.26E+00	9.05E+00	5.41E+00	4.76E-01	3.12E+00	1.62E+00	5.29E-01
9.53E-01	4.47E-01		2.67E-01	3.14E-01	2.55E-01	3.14E-01	9.98E-01	6.48E-01
3.94E-02	2.00E-02							3.85E-02
					4.59E-01		2.11E+00	2.27E+00
								5.60E+03
								2.28E+02
				1.68E+00	8.84E+01		4.17E+02	1.69E+01

**36**	red	3.13E-02		5.30E-02				6.27E-02		1.06E-01				9.40E-02		1.59E-01			
**37**	black			1.96E-01	8.95E-01					3.92E-01	1.79E+00					5.89E-01	2.69E+00		
**38**	purple	3.40E-02	9.77E-03	3.52E-02		6.68E+01		6.80E-02	1.95E-02	7.03E-02		1.34E+02		1.02E-01	2.93E-02	1.05E-01		2.01E+02	
**39**	blue		1.02E-02	2.35E-01	4.18E-01	3.76E+01	1.12E-02		2.05E-02	4.70E-01	8.36E-01	7.51E+01	2.23E-02		3.07E-02	7.05E-01	1.25E+00	1.13E+02	3.35E-02
**40**	brown	3.66E-02	3.09E-02	5.36E-02		2.35E-01	8.27E-03	7.32E-02	6.18E-02	1.07E-01		4.70E-01	1.65E-02	1.10E-01	9.27E-02	1.61E-01		7.04E-01	2.48E-02
**41**	pink		1.11E-02	5.06E-01	1.10E+00	2.86E-01	6.97E-03		2.22E-02	1.01E+00	2.21E+00	5.72E-01	1.39E-02		3.33E-02	1.52E+00	3.31E+00	8.58E-01	2.09E-02

** Information provided by the manufacturer on the ink packaging – „May contain trace amounts of Nickel”.

Table S10 Hazard Quotient (HQ) calculated for the analyzed elements contained in tattoo inks used to create a 5 cm tattoo using the realism method.

No	Product purpose	Color	Brand	Comment	5 cm – Realism MIN										
					Pb	Cd	Zn	Cr	Ni	Cu	As	Co	Sb	Se	Mn
1	tattoo ink	white	1	**				1.50E-01	2.79E-01	7.32E-01	3.30E-02		2.56E+03		
2		brown		**			7.41E-02	8.82E-01	1.04E+00	3.99E-01	2.63E-02	1.81E+00	3.80E+03	1.42E+02	6.06E+00
3		black				6.20E-02		1.85E-01	5.06E-01						
4		red						5.75E-02							
5		purple						1.13E-01	3.07E-01	9.77E+00	1.93E-02				
6		blue					3.00E-02	8.91E-02		4.59E+02					
7		orange		**				1.36E-01	3.59E-01	5.95E-01	1.48E-02				
8		pink		**				1.17E-01		6.57E-01	2.82E-02		2.18E+03		

No	Product purpose	Color	Brand	Comment	5 cm – Realism TYPICALLY										
					Pb	Cd	Zn	Cr	Ni	Cu	As	Co	Sb	Se	Mn
1	tattoo ink	white	1	**				3.01E-01	5.59E-01	1.46E+00	6.60E-02		5.11E+03		
2		brown		**			1.48E-01	1.76E+00	2.09E+00	7.98E-01	5.26E-02	3.62E+00	7.59E+03	2.83E+02	1.21E+01
3		black				1.24E-01		3.69E-01	1.01E+00						
4		red						1.15E-01							
5		purple						2.26E-01	6.15E-01	1.95E+01	3.86E-02				
6		blue					5.99E-02	1.78E-01		9.18E+02					
7		orange		**				2.72E-01	7.18E-01	1.19E+00	2.96E-02				
8		pink		**				2.33E-01		1.31E+00	5.64E-02		4.37E+03		

No	Product purpose	Color	Brand	Comment	5 cm – Realism MAX										
					Pb	Cd	Zn	Cr	Ni	Cu	As	Co	Sb	Se	Mn
1	tattoo ink	white	1	**				4.51E-01	8.38E-01	2.20E+00	9.90E-02		7.67E+03		
2		brown		**			2.22E-01	2.65E+00	3.13E+00	1.20E+00	7.89E-02	5.43E+00	1.14E+04	4.25E+02	1.82E+01
3		black				1.86E-01		5.54E-01	1.52E+00						
4		red						1.73E-01							
5		purple						3.39E-01	9.22E-01	2.93E+01	5.79E-02				
6		blue					8.99E-02	2.67E-01		1.38E+03					
7		orange		**				4.07E-01	1.08E+00	1.78E+00	4.44E-02				
8		pink		**				3.50E-01		1.97E+00	8.45E-02		6.55E+03		

**9**	green					1.05E-01	3.61E-01	1.15E+02	1.67E-02				
**10**	yellow					5.82E-02							
**11**	brown		7.07E-02		1.36E-01	1.29E+00	1.36E+00	4.67E+00	2.16E-02	1.19E+00	2.89E+03	1.02E+02	2.77E+01
**12**	black				4.69E-02	1.40E+00	4.43E+00	1.70E-01					1.23E+00
**13**	red					1.45E-01		4.03E+00					
**14**	purple	**2**			3.65E-02	1.02E+00	4.34E+00	6.71E-01					
**15**	blue					8.89E-02		6.15E+01	2.43E-02		2.24E+03		
**16**	pink					9.45E-02		6.30E-01	2.78E-02		2.26E+03		
**17**	green					7.51E-02		9.82E+01					

**9**	green					2.10E-01	7.23E-01	2.30E+02	3.34E-02				
**10**	yellow					1.16E-01							
**11**	brown		1.41E-01		2.71E-01	2.58E+00	2.72E+00	9.34E+00	4.32E-02	2.38E+00	5.78E+03	2.05E+02	5.54E+01
**12**	black				9.39E-02	2.79E+00	8.86E+00	3.39E-01					2.46E+00
**13**	red					2.89E-01		8.06E+00					
**14**	purple	**2**			7.30E-02	2.04E+00	8.69E+00	1.34E+00					
**15**	blue					1.78E-01		1.23E+02	4.86E-02		4.48E+03		
**16**	pink					1.89E-01		1.26E+00	5.56E-02		4.52E+03		
**17**	green					1.50E-01		1.96E+02					

**9**	green					3.16E-01	1.08E+00	3.45E+02	5.02E-02				
**10**	yellow					1.74E-01							
**11**	brown		2.12E-01		4.07E-01	3.87E+00	4.08E+00	1.40E+01	6.48E-02	3.57E+00	8.67E+03	3.07E+02	8.30E+01
**12**	black				1.41E-01	4.19E+00	1.33E+01	5.09E-01					3.70E+00
**13**	red					4.34E-01		1.21E+01					
**14**	purple	**2**			1.09E-01	3.06E+00	1.30E+01	2.01E+00					
**15**	blue					2.67E-01		1.84E+02	7.29E-02		6.72E+03		
**16**	pink					2.83E-01		1.89E+00	8.34E-02		6.77E+03		
**17**	green					2.25E-01		2.95E+02					

26	25	24	23	22	21	20	19	18
green	pink	orange	blue	purple	black	white	orange	yellow
			3					
		5.17E-02						1.19E-01
6.61E-02			6.29E-02					
	2.21E-02	2.68E-02			5.60E-02	1.96E-02		4.86E-02
8.98E-02	7.08E-01	1.96E+00	1.52E-01	8.53E-02	1.09E+00	9.34E-02	7.04E-02	1.52E+00
	7.40E+00	4.86E+00			4.44E+00			4.07E+00
2.38E+01	6.64E-01	2.36E-01	9.89E+01	7.71E-01	1.81E-01	7.62E-01	2.26E-01	2.14E-01
2.12E-02	2.21E-02		1.98E-02	2.04E-02		6.38E-02		
		8.47E-02						
						3.24E+03		
		2.30E+00						1.65E+00
		1.03E-01						2.38E-01
1.32E-01			1.26E-01					
	4.42E-02	5.35E-02			1.12E-01	3.92E-02		9.73E-02
1.80E-01	1.42E+00	3.91E+00	3.03E-01	1.71E-01	2.17E+00	1.87E-01	1.41E-01	3.05E+00
	1.48E+01	9.72E+00			8.88E+00			8.14E+00
4.77E+01	1.33E+00	4.71E-01	1.98E+02	1.54E+00	3.63E-01	1.52E+00	4.53E-01	4.28E-01
4.24E-02	4.42E-02		3.97E-02	4.09E-02		1.28E-01		
		1.69E-01						
						6.48E+03		
		4.61E+00						3.31E+00
		1.55E-01						3.58E-01
1.98E-01			1.89E-01					
	6.63E-02	8.03E-02			1.68E-01	5.88E-02		1.46E-01
2.69E-01	2.12E+00	5.87E+00	4.55E-01	2.56E-01	3.26E+00	2.80E-01	2.11E-01	4.57E+00
	2.22E+01	1.46E+01			1.33E+01			1.22E+01
7.15E+01	1.99E+00	7.07E-01	2.97E+02	2.31E+00	5.44E-01	2.29E+00	6.79E-01	6.42E-01
6.36E-02	6.64E-02		5.95E-02	6.13E-02		1.91E-01		
		2.54E-01						
						9.71E+03		
		6.91E+00						4.96E+00

	35	34	33	32	31	30	29	28	27
	tattoo & permanent makeup ink			permanent makeup ink					
	yellow	white	orange	brown-black	black coffee	chocolate-brown	black	chocolate	yellow
		7		6		5	4		
							4.66E-01	1.30E-01	
				6.95E-02	6.69E-02	1.30E-01	2.42E-01		
				1.18E-01	3.87E-02	9.46E-02	5.22E-02	4.81E-01	4.43E-01
	6.35E-01	9.52E-02	5.67E-01	8.64E-01	1.16E+00	9.86E-02	8.46E-01	3.82E-01	6.16E-01
	1.08E+00		8.39E-01	6.03E+00	3.61E+00	3.18E-01	2.08E+00	1.08E+00	3.53E-01
	6.35E-01	2.98E-01		1.78E-01	2.09E-01	1.70E-01	2.09E-01	6.65E-01	4.32E-01
	2.63E-02	1.34E-02							2.57E-02
						3.06E-01		1.40E+00	1.51E+00
									3.73E+03
									1.52E+02
					1.12E+00	5.90E+01		2.78E+02	1.13E+01
							9.32E-01	2.60E-01	
				1.39E-01	1.34E-01	2.59E-01	4.85E-01		
				2.35E-01	7.74E-02	1.89E-01	1.04E-01	9.63E-01	8.87E-01
	1.27E+00	1.90E-01	1.13E+00	1.73E+00	2.32E+00	1.97E-01	1.69E+00	7.64E-01	1.23E+00
	2.15E+00		1.68E+00	1.21E+01	7.22E+00	6.35E-01	4.16E+00	2.16E+00	7.05E-01
	1.27E+00	5.95E-01		3.57E-01	4.18E-01	3.40E-01	4.19E-01	1.33E+00	8.65E-01
	5.25E-02	2.67E-02							5.14E-02
						6.12E-01		2.81E+00	3.02E+00
									7.46E+03
									3.04E+02
					2.23E+00	1.18E+02		5.56E+02	2.26E+01
							1.40E+00	3.89E-01	
				2.09E-01	2.01E-01	3.89E-01	7.27E-01		
				3.53E-01	1.16E-01	2.84E-01	1.57E-01	1.44E+00	1.33E+00
	1.90E+00	2.85E-01	1.70E+00	2.59E+00	3.48E+00	2.96E-01	2.54E+00	1.15E+00	1.85E+00
	3.23E+00		2.52E+00	1.81E+01	1.08E+01	9.53E-01	6.24E+00	3.24E+00	1.06E+00
	1.91E+00	8.93E-01		5.35E-01	6.27E-01	5.10E-01	6.28E-01	2.00E+00	1.30E+00
	7.88E-02	4.01E-02							7.71E-02
						9.18E-01		4.21E+00	4.54E+00
									1.12E+04
									4.57E+02
					3.35E+00	1.77E+02		8.34E+02	3.38E+01

**36**	red	6.27E-02		1.06E-01				1.25E-01		2.12E-01				1.88E-01		3.18E-01			
**37**	black			3.92E-01	1.79E+00					7.85E-01	3.58E+00					1.18E+00	5.37E+00		
**38**	purple	6.80E-02	1.95E-02	7.03E-02		1.34E+02		1.36E-01	3.91E-02	1.41E-01		2.67E+02		2.04E-01	5.86E-02	2.11E-01		4.01E+02	
**39**	blue		2.05E-02	4.70E-01	8.36E-01	7.51E+01	2.23E-02		4.10E-02	9.40E-01	1.67E+00	1.50E+02	4.47E-02		6.15E-02	1.41E+00	2.51E+00	2.25E+02	6.70E-02
**40**	brown	7.32E-02	6.18E-02	1.07E-01		4.70E-01	1.65E-02	1.46E-01	1.24E-01	2.14E-01		9.39E-01	3.31E-02	2.20E-01	1.85E-01	3.21E-01		1.41E+00	4.96E-02
**41**	pink		2.22E-02	1.01E+00	2.21E+00	5.72E-01	1.39E-02		4.43E-02	2.03E+00	4.42E+00	1.14E+00	2.79E-02		6.65E-02	3.04E+00	6.63E+00	1.72E+00	4.18E-02

** Information provided by the manufacturer on the ink packaging – „May contain trace amounts of Nickel”.

Table S11 Hazard Quotient (HQ) calculated for the analyzed elements contained in tattoo inks used to create a 5 cm tattoo using the filling method.

No	Product purpose	Color	Brand	Comment	5 cm – Filling MIN										
					Pb	Cd	Zn	Cr	Ni	Cu	As	Co	Sb	Se	Mn
1	tattoo ink	white	1	**				2.26E-01	4.19E-01	1.10E+00	4.95E-02		3.83E+03		
2		brown		**			1.11E-01	1.32E+00	1.57E+00	5.98E-01	3.95E-02	2.71E+00	5.70E+03	2.13E+02	9.09E+00
3		black				9.31E-02		2.77E-01	7.59E-01						
4		red						8.63E-02							
5		purple						1.69E-01	4.61E-01	1.47E+01	2.90E-02				
6		blue					4.49E-02	1.34E-01		6.88E+02					
7		orange		**				2.04E-01	5.38E-01	8.92E-01	2.22E-02				
8		pink		**				1.75E-01		9.85E-01	4.23E-02		3.27E+03		

No	Product purpose	Color	Brand	Comment	5 cm – Filling TYPICALLY										
					Pb	Cd	Zn	Cr	Ni	Cu	As	Co	Sb	Se	Mn
1	tattoo ink	white	1	**				4.51E-01	8.38E-01	2.20E+00	9.90E-02		7.67E+03		
2		brown		**			2.22E-01	2.65E+00	3.13E+00	1.20E+00	7.89E-02	5.43E+00	1.14E+04	4.25E+02	1.82E+01
3		black				1.86E-01		5.54E-01	1.52E+00						
4		red						1.73E-01							
5		purple						3.39E-01	9.22E-01	2.93E+01	5.79E-02				
6		blue					8.99E-02	2.67E-01		1.38E+03					
7		orange		**				4.07E-01	1.08E+00	1.78E+00	4.44E-02				
8		pink		**				3.50E-01		1.97E+00	8.45E-02		6.55E+03		

No	Product purpose	Color	Brand	Comment	5 cm – Filling MAX										
					Pb	Cd	Zn	Cr	Ni	Cu	As	Co	Sb	Se	Mn
1	tattoo ink	white	1	**				6.77E-01	1.26E+00	3.30E+00	1.48E-01		1.15E+04		
2		brown		**			3.34E-01	3.97E+00	4.70E+00	1.79E+00	1.18E-01	8.14E+00	1.71E+04	6.38E+02	2.73E+01
3		black				2.79E-01		8.31E-01	2.28E+00						
4		red						2.59E-01							
5		purple						5.08E-01	1.38E+00	4.40E+01	8.69E-02				
6		blue					1.35E-01	4.01E-01		2.07E+03					
7		orange		**				6.11E-01	1.61E+00	2.68E+00	6.66E-02				
8		pink		**				5.24E-01		2.96E+00	1.27E-01		9.82E+03		

17	16	15	14	13	12	11	10	9
green	pink	blue	purple	red	black	brown	yellow	green
			2					
						1.06E-01		
			5.47E-02		7.04E-02	2.04E-01		
1.13E-01	1.42E-01	1.33E-01	1.53E+00	2.17E-01	2.09E+00	1.94E+00	8.72E-02	1.58E-01
			6.52E+00		6.64E+00	2.04E+00		5.42E-01
1.47E+02	9.45E-01	9.22E+01	1.01E+00	6.05E+00	2.55E-01	7.01E+00		1.72E+02
	4.17E-02	3.65E-02				3.24E-02		2.51E-02
						1.78E+00		
	3.39E+03	3.36E+03				4.34E+03		
						1.54E+02		
					1.85E+00	4.15E+01		
						2.12E-01		
			1.09E-01		1.41E-01	4.07E-01		
2.25E-01	2.83E-01	2.67E-01	3.06E+00	4.34E-01	4.19E+00	3.87E+00	1.74E-01	3.16E-01
			1.30E+01		1.33E+01	4.08E+00		1.08E+00
2.95E+02	1.89E+00	1.84E+02	2.01E+00	1.21E+01	5.09E-01	1.40E+01		3.45E+02
	8.34E-02	7.29E-02				6.48E-02		5.02E-02
						3.57E+00		
	6.77E+03	6.72E+03				8.67E+03		
						3.07E+02		
					3.70E+00	8.30E+01		
						3.18E-01		
			1.64E-01		2.11E-01	6.11E-01		
3.38E-01	4.25E-01	4.00E-01	4.59E+00	6.51E-01	6.28E+00	5.81E+00	2.62E-01	4.73E-01
			1.95E+01		1.99E+01	6.11E+00		1.63E+00
4.42E+02	2.83E+00	2.77E+02	3.02E+00	1.81E+01	7.64E-01	2.10E+01		5.17E+02
	1.25E-01	1.09E-01				9.72E-02		7.53E-02
						5.35E+00		
	1.02E+04	1.01E+04				1.30E+04		
						4.61E+02		
					5.54E+00	1.25E+02		

26	25	24	23	22	21	20	19	18
green	pink	orange	blue	purple	black	white	orange	yellow
			3					
		7.75E-02						1.79E-01
9.91E-02			9.43E-02					
	3.32E-02	4.01E-02			8.39E-02	2.94E-02		7.30E-02
1.35E-01	1.06E+00	2.93E+00	2.27E-01	1.28E-01	1.63E+00	1.40E-01	1.06E-01	2.28E+00
	1.11E+01	7.29E+00			6.66E+00			6.11E+00
3.58E+01	9.97E-01	3.54E-01	1.48E+02	1.16E+00	2.72E-01	1.14E+00	3.40E-01	3.21E-01
3.18E-02	3.32E-02		2.98E-02	3.06E-02		9.57E-02		
		1.27E-01						
						4.86E+03		
		3.46E+00						2.48E+00
		1.55E-01						3.58E-01
1.98E-01			1.89E-01					
	6.63E-02	8.03E-02			1.68E-01	5.88E-02		1.46E-01
2.69E-01	2.12E+00	5.87E+00	4.55E-01	2.56E-01	3.26E+00	2.80E-01	2.11E-01	4.57E+00
	2.22E+01	1.46E+01			1.33E+01			1.22E+01
7.15E+01	1.99E+00	7.07E-01	2.97E+02	2.31E+00	5.44E-01	2.29E+00	6.79E-01	6.42E-01
6.36E-02	6.64E-02		5.95E-02	6.13E-02		1.91E-01		
		2.54E-01						
						9.71E+03		
		6.91E+00						4.96E+00
		2.32E-01						5.36E-01
2.97E-01			2.83E-01					
	9.95E-02	1.20E-01			2.52E-01	8.82E-02		2.19E-01
4.04E-01	3.19E+00	8.80E+00	6.82E-01	3.84E-01	4.88E+00	4.20E-01	3.17E-01	6.85E+00
	3.33E+01	2.19E+01			2.00E+01			1.83E+01
1.07E+02	2.99E+00	1.06E+00	4.45E+02	3.47E+00	8.16E-01	3.43E+00	1.02E+00	9.63E-01
9.54E-02	9.95E-02		8.93E-02	9.19E-02		2.87E-01		
		3.81E-01				0.00E+00		
						1.46E+04		
		1.04E+01						7.44E+00

35	34	33	32	31	30	29	28	27
tattoo & permanent makeup ink			permanent makeup ink					
yellow	white	orange	brown-black	black coffee	chocolate-brown	black	chocolate	yellow
	7		6		5	4		
						6.99E-01	1.95E-01	
			1.04E-01	1.00E-01	1.95E-01	3.64E-01		
			1.77E-01	5.80E-02	1.42E-01	7.83E-02	7.22E-01	6.65E-01
9.52E-01	1.43E-01	8.50E-01	1.30E+00	1.74E+00	1.48E-01	1.27E+00	5.73E-01	9.24E-01
1.61E+00		1.26E+00	9.05E+00	5.41E+00	4.76E-01	3.12E+00	1.62E+00	5.29E-01
9.53E-01	4.47E-01		2.67E-01	3.14E-01	2.55E-01	3.14E-01	9.98E-01	6.48E-01
3.94E-02	2.00E-02							3.85E-02
					4.59E-01		2.11E+00	2.27E+00
								5.60E+03
								2.28E+02
				1.68E+00	8.84E+01		4.17E+02	1.69E+01
						1.40E+00	3.89E-01	
			2.09E-01	2.01E-01	3.89E-01	7.27E-01		
			3.53E-01	1.16E-01	2.84E-01	1.57E-01	1.44E+00	1.33E+00
1.90E+00	2.85E-01	1.70E+00	2.59E+00	3.48E+00	2.96E-01	2.54E+00	1.15E+00	1.85E+00
3.23E+00		2.52E+00	1.81E+01	1.08E+01	9.53E-01	6.24E+00	3.24E+00	1.06E+00
1.91E+00	8.93E-01		5.35E-01	6.27E-01	5.10E-01	6.28E-01	2.00E+00	1.30E+00
7.88E-02	4.01E-02							7.71E-02
					9.18E-01		4.21E+00	4.54E+00
								1.12E+04
								4.57E+02
				3.35E+00	1.77E+02		8.34E+02	3.38E+01
						2.10E+00	5.84E-01	
			3.13E-01	3.01E-01	5.84E-01	1.09E+00		
			5.30E-01	1.74E-01	4.26E-01	2.35E-01	2.17E+00	2.00E+00
2.86E+00	4.28E-01	2.55E+00	3.89E+00	5.23E+00	4.44E-01	3.81E+00	1.72E+00	2.77E+00
4.84E+00		3.78E+00	2.71E+01	1.62E+01	1.43E+00	9.37E+00	4.86E+00	1.59E+00
2.86E+00	1.34E+00		8.02E-01	9.41E-01	7.65E-01	9.42E-01	2.99E+00	1.95E+00
1.18E-01	6.01E-02							1.16E-01
					1.38E+00		6.32E+00	6.81E+00
								1.68E+04
								6.85E+02
				5.03E+00	2.65E+02		1.25E+03	5.07E+01

**36**	red	9.40E-02		1.59E-01				1.88E-01		3.18E-01				2.82E-01		4.77E-01			
**37**	black			5.89E-01	2.69E+00					1.18E+00	5.37E+00					1.77E+00	8.06E+00		
**38**	purple	1.02E-01	2.93E-02	1.05E-01		2.01E+02		2.04E-01	5.86E-02	2.11E-01		4.01E+02		3.06E-01	8.79E-02	3.16E-01		6.02E+02	
**39**	blue		3.07E-02	7.05E-01	1.25E+00	1.13E+02	3.35E-02		6.15E-02	1.41E+00	2.51E+00	2.25E+02	6.70E-02		9.22E-02	2.12E+00	3.76E+00	3.38E+02	1.01E-01
**40**	brown	1.10E-01	9.27E-02	1.61E-01		7.04E-01	2.48E-02	2.20E-01	1.85E-01	3.21E-01		1.41E+00	4.96E-02	3.29E-01	2.78E-01	4.82E-01		2.11E+00	7.45E-02
**41**	pink		3.33E-02	1.52E+00	3.31E+00	8.58E-01	2.09E-02		6.65E-02	3.04E+00	6.63E+00	1.72E+00	4.18E-02		9.98E-02	4.56E+00	9.94E+00	2.57E+00	6.27E-02

** Information provided by the manufacturer on the ink packaging – „May contain trace amounts of Nickel”.

Table S12 Lifetime Cancer Risk (LCR) calculated for the analyzed elements contained in tattoo inks used to create a 5 cm tattoo using the contour method.

No	Product purpose	Color	Brand	Comments	5 cm – Contour MIN					5 cm – Contour TYPICALLY					5 cm – Contour MAX				
					Pb	Cd	Cr	Ni	As	Pb	Cd	Cr	Ni	As	Pb	Cd	Cr	Ni	As
1	tattoo ink	white	1	**			2.51E-10	2.63E-06	2.78E-08			5.01E-10	5.26E-06	5.57E-08			7.52E-10	7.89E-06	8.35E-08
2		brown		**			1.47E-09	9.82E-06	2.22E-08			2.94E-09	1.96E-05	4.44E-08			4.41E-09	2.95E-05	6.66E-08
3		black				8.16E-11	3.08E-10	4.76E-06			1.63E-10	6.15E-10	9.52E-06			2.45E-10	9.23E-10	1.43E-05	
4		red					9.58E-11					1.92E-10					2.88E-10		
5		purple					1.88E-10	2.89E-06	1.63E-08			3.77E-10	5.78E-06	3.26E-08			5.65E-10	8.68E-06	4.89E-08
6		blue					1.48E-10					2.97E-10					4.45E-10		
7		orange		**			2.26E-10	3.38E-06	1.25E-08			4.53E-10	6.75E-06	2.50E-08			6.79E-10	1.01E-05	3.74E-08
8		pink		**			1.94E-10		2.38E-08			3.88E-10		4.76E-08			5.83E-10		7.13E-08

**9**	green			1.75E-10	3.40E-06	1.41E-08		3.51E-10	6.80E-06	2.82E-08		5.26E-10	1.02E-05	4.23E-08
**10**	yellow			9.69E-11				1.94E-10				2.91E-10		
**11**	brown		1.50E-07	2.15E-09	1.28E-05	1.82E-08	2.99E-07	4.30E-09	2.56E-05	3.64E-08	4.49E-07	6.45E-09	3.84E-05	5.47E-08
**12**	black			2.33E-09	4.17E-05			4.66E-09	8.34E-05			6.98E-09	1.25E-04	
**13**	red			2.41E-10				4.82E-10				7.24E-10		
**14**	purple	**2**		1.70E-09	4.09E-05			3.40E-09	8.18E-05			5.10E-09	1.23E-04	
**15**	blue			1.48E-10		2.05E-08		2.96E-10		4.10E-08		4.45E-10		6.15E-08
**16**	pink			1.57E-10		2.34E-08		3.15E-10		4.69E-08		4.72E-10		7.03E-08
**17**	green			1.25E-10				2.50E-10				3.75E-10		

**18**	yellow		2.52E-07		2.54E-09	3.83E-05		5.05E-07		5.08E-09	7.66E-05		7.57E-07		7.61E-09	1.15E-04	
**19**	orange				1.17E-10					2.35E-10					3.52E-10		
**20**	white				1.56E-10		5.38E-08			3.11E-10		1.08E-07			4.67E-10		1.61E-07
**21**	black				1.81E-09	4.18E-05				3.62E-09	8.35E-05				5.43E-09	1.25E-04	
**22**	purple				1.42E-10		1.72E-08			2.84E-10		3.45E-08			4.27E-10		5.17E-08
**23**	blue	**3**		8.27E-11	2.53E-10		1.67E-08		1.65E-10	5.05E-10		3.35E-08		2.48E-10	7.58E-10		5.02E-08
**24**	orange		1.09E-07		3.26E-09	4.57E-05		2.19E-07		6.52E-09	9.15E-05		3.28E-07		9.78E-09	1.37E-04	
**25**	pink				1.18E-09	6.97E-05	1.87E-08			2.36E-09	1.39E-04	3.73E-08			3.54E-09	2.09E-04	5.60E-08
**26**	green			8.69E-11	1.50E-10		1.79E-08		1.74E-10	2.99E-10		3.58E-08		2.61E-10	4.49E-10		5.36E-08

**27**		yellow				1.03E-09	3.32E-06	2.17E-08			2.05E-09	6.64E-06	4.34E-08			3.08E-09	9.96E-06	6.50E-08
**28**	**permanent makeup ink**	chocolate	**4**	2.75E-07		6.37E-10	1.02E-05		5.50E-07		1.27E-09	2.03E-05		8.25E-07		1.91E-09	3.05E-05	
**29**		black		9.87E-07	3.19E-10	1.41E-09	1.96E-05		1.97E-06	6.38E-10	2.82E-09	3.92E-05		2.96E-06	9.57E-10	4.23E-09	5.88E-05	
**30**		chocolate-brown	**5**		1.71E-10	1.64E-10	2.99E-06			3.41E-10	3.29E-10	5.98E-06			5.12E-10	4.93E-10	8.97E-06	
**31**		black coffee	**6**		8.80E-11	1.94E-09	3.40E-05			1.76E-10	3.87E-09	6.79E-05			2.64E-10	5.81E-09	1.02E-04	
**32**		brown-black			9.15E-11	1.44E-09	5.68E-05			1.83E-10	2.88E-09	1.14E-04			2.74E-10	4.32E-09	1.70E-04	
**33**	**tattoo & permanent makeup ink**	orange	**7**			9.44E-10	7.90E-06				1.89E-09	1.58E-05				2.83E-09	2.37E-05	
**34**		white				1.59E-10		1.13E-08			3.17E-10		2.25E-08			4.76E-10		3.38E-08
**35**		yellow				1.06E-09	1.01E-05	2.22E-08			2.12E-09	2.02E-05	4.43E-08			3.17E-09	3.04E-05	6.65E-08

**36**	red	8.25E-11	1.77E-10			1.65E-10	3.54E-10			2.47E-10	5.30E-10		
**37**	black		6.54E-10	1.69E-05			1.31E-09	3.37E-05			1.96E-09	5.06E-05	
**38**	purple	8.94E-11	1.17E-10			1.79E-10	2.34E-10			2.68E-10	3.52E-10		
**39**	blue		7.83E-10	7.87E-06	1.88E-08		1.57E-09	1.57E-05	3.77E-08		2.35E-09	2.36E-05	5.65E-08
**40**	brown	9.63E-11	1.79E-10		1.40E-08	1.93E-10	3.57E-10		2.79E-08	2.89E-10	5.36E-10		4.19E-08
**41**	pink		1.69E-09	2.08E-05	1.18E-08		3.38E-09	4.16E-05	2.35E-08		5.06E-09	6.24E-05	3.53E-08

** Information provided by the manufacturer on the ink packaging – „May contain trace amounts of Nickel”.

Table S13 Lifetime Cancer Risk (LCR) calculated for the analyzed elements contained in tattoo inks used to create a 5 cm tattoo using the realism method.

No	Product purpose	Color	Brand	Comments	5 cm – Realism MIN					5 cm – Realism TYPICALLY					5 cm – Realism MAX				
					Pb	Cd	Cr	Ni	As	Pb	Cd	Cr	Ni	As	Pb	Cd	Cr	Ni	As
1	tattoo ink	white	1	**			5.01E-10	5.26E-06	5.57E-08			1.00E-09	1.05E-05	1.11E-07			1.50E-09	1.58E-05	1.67E-07
2		brown		**			2.94E-09	1.96E-05	4.44E-08			5.88E-09	3.93E-05	8.88E-08			8.82E-09	5.89E-05	1.33E-07
3		black				1.63E-10	6.15E-10	9.52E-06			3.27E-10	1.23E-09	1.90E-05			4.90E-10	1.85E-09	2.86E-05	
4		red					1.92E-10					3.83E-10					5.75E-10		
5		purple					3.77E-10	5.78E-06	3.26E-08			7.53E-10	1.16E-05	6.52E-08			1.13E-09	1.74E-05	9.78E-08
6		blue					2.97E-10					5.94E-10					8.91E-10		
7		orange		**			4.53E-10	6.75E-06	2.50E-08			9.06E-10	1.35E-05	4.99E-08			1.36E-09	2.03E-05	7.49E-08
8		pink		**			3.88E-10		4.76E-08			7.77E-10		9.51E-08			1.17E-09		1.43E-07

**9**	green			3.51E-10	6.80E-06	2.82E-08		7.01E-10	1.36E-05	5.64E-08		1.05E-09	2.04E-05	8.47E-08
**10**	yellow			1.94E-10				3.88E-10				5.82E-10		
**11**	brown		2.99E-07	4.30E-09	2.56E-05	3.64E-08	5.98E-07	8.60E-09	5.12E-05	7.29E-08	8.98E-07	1.29E-08	7.67E-05	1.09E-07
**12**	black			4.66E-09	8.34E-05			9.31E-09	1.67E-04			1.40E-08	2.50E-04	
**13**	red			4.82E-10				9.65E-10				1.45E-09		
**14**	purple	**2**		3.40E-09	8.18E-05			6.80E-09	1.64E-04			1.02E-08	2.45E-04	
**15**	blue			2.96E-10		4.10E-08		5.93E-10		8.21E-08		8.89E-10		1.23E-07
**16**	pink			3.15E-10		4.69E-08		6.30E-10		9.38E-08		9.45E-10		1.41E-07
**17**	green			2.50E-10				5.01E-10				7.51E-10		

**18**	yellow		5.05E-07		5.08E-09	7.66E-05		1.01E-06		1.02E-08	1.53E-04		1.51E-06		1.52E-08	2.30E-04	
**19**	orange				2.35E-10					4.69E-10					7.04E-10		
**20**	white				3.11E-10		1.08E-07			6.23E-10		2.15E-07			9.34E-10		3.23E-07
**21**	black				3.62E-09	8.35E-05				7.24E-09	1.67E-04				1.09E-08	2.51E-04	
**22**	purple				2.84E-10		3.45E-08			5.69E-10		6.89E-08			8.53E-10		1.03E-07
**23**	blue	**3**		1.65E-10	5.05E-10		3.35E-08		3.31E-10	1.01E-09		6.70E-08		4.96E-10	1.52E-09		1.00E-07
**24**	orange		2.19E-07		6.52E-09	9.15E-05		4.38E-07		1.30E-08	1.83E-04		6.56E-07		1.96E-08	2.74E-04	
**25**	pink				2.36E-09	1.39E-04	3.73E-08			4.72E-09	2.79E-04	7.47E-08			7.08E-09	4.18E-04	1.12E-07
**26**	green			1.74E-10	2.99E-10		3.58E-08		3.48E-10	5.98E-10		7.15E-08		5.22E-10	8.98E-10		1.07E-07

35	34	33	32	31	30	29	28	27
tattoo & permanent makeup ink			permanent makeup ink					
yellow	white	orange	brown-black	black coffee	chocolate-brown	black	chocolate	yellow
	7		6		5	4		
						1.97E-06	5.50E-07	
			1.83E-10	1.76E-10	3.41E-10	6.38E-10		
2.12E-09	3.17E-10	1.89E-09	2.88E-09	3.87E-09	3.29E-10	2.82E-09	1.27E-09	2.05E-09
2.02E-05		1.58E-05	1.14E-04	6.79E-05	5.98E-06	3.92E-05	2.03E-05	6.64E-06
4.43E-08	2.25E-08							4.34E-08
						3.95E-06	1.10E-06	
			3.66E-10	3.52E-10	6.83E-10	1.28E-09		
4.23E-09	6.34E-10	3.78E-09	5.76E-09	7.74E-09	6.57E-10	5.64E-09	2.55E-09	4.11E-09
4.05E-05		3.16E-05	2.27E-04	1.36E-04	1.20E-05	7.83E-05	4.07E-05	1.33E-05
8.86E-08	4.51E-08							8.67E-08
						5.92E-06	1.65E-06	
			5.49E-10	5.28E-10	1.02E-09	1.91E-09		
6.35E-09	9.52E-10	5.67E-09	8.64E-09	1.16E-08	9.86E-10	8.46E-09	3.82E-09	6.16E-09
6.07E-05		4.74E-05	3.41E-04	2.04E-04	1.79E-05	1.18E-04	6.10E-05	1.99E-05
1.33E-07	6.76E-08							1.30E-07

**36**	red	1.65E-10	3.54E-10			3.30E-10	7.07E-10			4.95E-10	1.06E-09		
**37**	black		1.31E-09	3.37E-05			2.62E-09	6.74E-05			3.92E-09	1.01E-04	
**38**	purple	1.79E-10	2.34E-10			3.58E-10	4.69E-10			5.37E-10	7.03E-10		
**39**	blue		1.57E-09	1.57E-05	3.77E-08		3.13E-09	3.15E-05	7.54E-08		4.70E-09	4.72E-05	1.13E-07
**40**	brown	1.93E-10	3.57E-10		2.79E-08	3.85E-10	7.14E-10		5.58E-08	5.78E-10	1.07E-09		8.38E-08
**41**	pink		3.38E-09	4.16E-05	2.35E-08		6.75E-09	8.32E-05	4.71E-08		1.01E-08	1.25E-04	7.06E-08

** Information provided by the manufacturer on the ink packaging – „May contain trace amounts of Nickel".

Table S14 Lifetime Cancer Risk (LCR) calculated for the analyzed elements contained in tattoo inks used to create a 5 cm tattoo using the filling method.

No	Product purpose	Color	Brand	Comments	5 cm – Filling MIN					5 cm – Filling TYPICALLY					5 cm – Filling MAX				
					Pb	Cd	Cr	Ni	As	Pb	Cd	Cr	Ni	As	Pb	Cd	Cr	Ni	As
1	tattoo ink	white	1	**			6.79E-10	1.01E-05	3.74E-08			1.36E-09	2.03E-05	7.49E-08			2.04E-09	3.04E-05	1.12E-07
2		brown		**			4.41E-09	2.95E-05	6.66E-08			8.82E-09	5.89E-05	1.33E-07			1.32E-08	8.84E-05	2.00E-07
3		black					5.65E-10	8.68E-06	4.89E-08			1.13E-09	1.74E-05	9.78E-08			1.69E-09	2.60E-05	1.47E-07
4		red					5.26E-10	1.02E-05	4.23E-08			1.05E-09	2.04E-05	8.47E-08			1.58E-09	3.06E-05	1.27E-07
5		purple					2.88E-10					5.75E-10					8.63E-10		
6		blue					2.91E-10					5.82E-10					8.72E-10		
7		orange		**		2.45E-10	9.23E-10	1.43E-05			4.90E-10	1.85E-09	2.86E-05			7.35E-10	2.77E-09	4.29E-05	
8		pink		**			5.83E-10		7.13E-08			1.17E-09		1.43E-07			1.75E-09		2.14E-07

**9**	green				4.45E-10					8.91E-10					1.34E-09		
**10**	yellow				7.52E-10	7.89E-06	8.35E-08			1.50E-09	1.58E-05	1.67E-07			2.26E-09	2.37E-05	2.51E-07
**11**	brown				4.45E-10		6.15E-08			8.89E-10		1.23E-07			1.33E-09		1.85E-07
**12**	black		7.57E-07		7.61E-09	1.15E-04		1.51E-06		1.52E-08	2.30E-04		2.27E-06		2.28E-08	3.45E-04	
**13**	red				3.75E-10					7.51E-10					1.13E-09		
**14**	purple	**2**			6.98E-09	1.25E-04				1.40E-08	2.50E-04				2.09E-08	3.75E-04	
**15**	blue				5.10E-09	1.23E-04				1.02E-08	2.45E-04				1.53E-08	3.68E-04	
**16**	pink		4.49E-07		6.45E-09	3.84E-05	5.47E-08	8.98E-07		1.29E-08	7.67E-05	1.09E-07	1.35E-06		1.94E-08	1.15E-04	1.64E-07
**17**	green				4.72E-10		7.03E-08			9.45E-10		1.41E-07			1.42E-09		2.11E-07

**18**	yellow				7.24E-10					1.45E-09					2.17E-09		
**19**	orange				3.52E-10					7.04E-10					1.06E-09		
**20**	white			2.61E-10	4.49E-10		5.36E-08		5.22E-10	8.98E-10		1.07E-07		7.82E-10	1.35E-09		1.61E-07
**21**	black				3.54E-09	2.09E-04	5.60E-08			7.08E-09	4.18E-04	1.12E-07			1.06E-08	6.27E-04	1.68E-07
**22**	purple				4.67E-10		1.61E-07			9.34E-10		3.23E-07			1.40E-09		4.84E-07
**23**	blue	**3**		2.48E-10	7.58E-10		5.02E-08		4.96E-10	1.52E-09		1.00E-07		7.44E-10	2.27E-09		1.51E-07
**24**	orange		3.28E-07		9.78E-09	1.37E-04		6.56E-07		1.96E-08	2.74E-04		9.85E-07		2.93E-08	4.12E-04	
**25**	pink				5.43E-09	1.25E-04				1.09E-08	2.51E-04				1.63E-08	3.76E-04	
**26**	green				4.27E-10		5.17E-08			8.53E-10		1.03E-07			1.28E-09		1.55E-07

27		yellow				3.08E-09	9.96E-06	6.50E-08			6.16E-09	1.99E-05	1.30E-07			9.24E-09	2.99E-05	1.95E-07
28	permanent makeup ink	chocolat e	4	8.25E-07		1.91E-09	3.05E-05		1.65E-06		3.82E-09	6.10E-05		2.47E-06		5.73E-09	9.15E-05	
29		black		2.96E-06	9.57E-10	4.23E-09	5.88E-05		5.92E-06	1.91E-09	8.46E-09	1.18E-04		8.88E-06	2.87E-09	1.27E-08	1.76E-04	
30		chocolat e-brown	5		5.12E-10	4.93E-10	8.97E-06			1.02E-09	9.86E-10	1.79E-05			1.54E-09	1.48E-09	2.69E-05	
31		black coffee	6		2.64E-10	5.81E-09	1.02E-04			5.28E-10	1.16E-08	2.04E-04			7.92E-10	1.74E-08	3.06E-04	
32		brown-black			2.74E-10	4.32E-09	1.70E-04			5.49E-10	8.64E-09	3.41E-04			8.23E-10	1.30E-08	5.11E-04	
33	tattoo & permanent makeup ink	orange	7			2.83E-09	2.37E-05				5.67E-09	4.74E-05				8.50E-09	7.11E-05	
34		white				4.76E-10		3.38E-08			9.52E-10		6.76E-08			1.43E-09		1.01E-07
35		yellow				3.17E-09	3.04E-05	6.65E-08			6.35E-09	6.07E-05	1.33E-07			9.52E-09	9.11E-05	1.99E-07

**36**	red	2.47E-10	5.30E-10			4.95E-10	1.06E-09			7.42E-10	1.59E-09		
**37**	black		1.96E-09	5.06E-05			3.92E-09	1.01E-04			5.89E-09	1.52E-04	
**38**	purple	2.68E-10	3.52E-10			5.37E-10	7.03E-10			8.05E-10	1.05E-09		
**39**	blue		2.35E-09	2.36E-05	5.65E-08		4.70E-09	4.72E-05	1.13E-07		7.05E-09	7.08E-05	1.70E-07
**40**	brown	2.89E-10	5.36E-10		4.19E-08	5.78E-10	1.07E-09		8.38E-08	8.67E-10	1.61E-09		1.26E-07
**41**	pink		5.06E-09	6.24E-05	3.53E-08		1.01E-08	1.25E-04	7.06E-08		1.52E-08	1.87E-04	1.06E-07

** Information provided by the manufacturer on the ink packaging – „May contain trace amounts of Nickel".
